# Fam3C alters Golgi apparatus morphology and function in triple negative breast cancer

**DOI:** 10.1093/jmcb/mjaf042

**Published:** 2025-11-17

**Authors:** Annamarie C Dalton, Elisabeth R M Rochel, William S Streitfeld, Cécile Fréreux, Breege V Howley, Philip H Howe

**Affiliations:** Department of Biochemistry and Molecular Biology, Medical University of South Carolina, Charleston, SC 29425, USA; Department of Biochemistry and Molecular Biology, Medical University of South Carolina, Charleston, SC 29425, USA; Department of Biochemistry and Molecular Biology, Medical University of South Carolina, Charleston, SC 29425, USA; Department of Cell, Developmental, and Cancer Biology, Oregon Health and Science University, Portland, OR 97239, USA; Department of Biochemistry and Molecular Biology, Medical University of South Carolina, Charleston, SC 29425, USA; Department of Biochemistry and Molecular Biology, Medical University of South Carolina, Charleston, SC 29425, USA; Department of Biochemistry and Molecular Biology, Medical University of South Carolina, Charleston, SC 29425, USA

**Keywords:** Fam3C, Golgi apparatus, ILEI, triple negative breast cancer, EMT

## Abstract

Fam3C, also known as ILEI, is an established regulator of the epithelial-to-mesenchymal transition (EMT) and breast cancer stem cell phenotypes. Multiple cancer cell models and orthotopic animal model experiments have demonstrated a role for Fam3C in tumor progression and metastasis. Here, we establish Fam3C’s impact on triple negative breast cancer (TNBC) patients and genetically engineered mouse models of spontaneous breast cancer tumor progression. Though Fam3C is a known secreted protein, we discovered its retention in the Golgi apparatus through anchoring of its signal peptide into the membrane before its signal peptide and pro-peptide are processed and removed. While retained in the Golgi apparatus, Fam3C affects the overall morphology of the organelle and its biological functions, including alterations in protein secretion and invasive potential. Expanding our knowledge of the biological mechanisms behind EMT will help develop therapies to specifically target cells with increased metastatic potential in TNBC.

## Introduction

Triple negative breast cancer (TNBC) is clinically challenging due to its aggressive and metastatic nature, high rate of recurrence, lack of clinically targeted receptors, and developed resistance to chemotherapies (Yin et al., 2020). Overall, there is a poor prognosis associated with TNBC diagnosis (Yin et al, 2020). Several characteristics of TNBC are in part attributed to the plastic nature of cells that have activated epithelial-to-mesenchymal transition (EMT) pathways. These plastic cells display properties of cancer stem cells (CSCs), including the capacity to self-renew and the characteristic resistance to chemotherapeutics (Tanabe et al., 2020). Understanding the mechanisms that drive EMT and CSC phenotypes will be critical to develop therapies that can prevent this transition to a deadly metastatic state. Fam3C is one established protein in EMT that promotes CSC properties. Fam3C is sometimes referred to by the acronym ILEI for interleukin-like EMT inducer. Several groups have published data that support its role in EMT and the development of metastatic lesions (Waerner et al., 2006; Csiszar et al., 2014; Woosley et al., 2019). Despite work over the past few decades, the mechanisms of Fam3C function are still under investigation.

Fam3C transgenic mice have illuminated a variety of physiological and pathological effects, linking Fam3C to the maintenance of bone homeostasis, liver fibrosis, and anemia (Määttä et al., 2016; Schmidt et al., 2023). Multiple orthotopic mouse models using various mouse and human cancer cell lines have established a role for Fam3C in cancer progression and metastasis (Waerner et al., 2006; Lahsnig et al., 2009; Csiszar et al., 2014; Woosley et al., 2019; Schmidt et al., 2021). To our knowledge, no spontaneous genetically engineered mouse models (GEMMs) of cancer progression have been used to study Fam3C to date. Analysis of human Fam3C protein in breast cancer patient microarray cores demonstrated a correlation between Fam3C protein expression, tumorigenesis, and metastatic lesions within the lymph node (Woosley et al., 2019). Several groups have established Fam3C’s role in which may explain its increased expression in metastatic cells. Waerner et al. (2006) demonstrated that in transformed mammary epithelial cells, Fam3C can drive changes in ERK signaling and regulate migration and invasion. The authors further demonstrated the increased metastatic burden in xenograft mouse models of Fam3C overexpression (Waerner et al., 2006). In human patient samples of colon and mammary carcinomas, Waerner et al. (2006) compared ‘granular’ Fam3C staining versus diffuse cytoplasmic staining and found that cytoplasmic staining correlated with decreased metastasis-free survival and overall survival.

Mechanistically, multiple regulators of Fam3C expression have been identified at both transcriptional and post-transcriptional levels in a variety of contexts (Pilipenko et al., 2004; Hussey et al., 2012; Song et al., 2014; Xue et al., 2014; Noguchi et al., 2018). Fam3C regulation has been studied in relation to Alzheimer’s disease, bone remodeling, stress response, and cancer progression (Hasegawa et al., 2014; Liu et al., 2016; Määttä et al., 2016; Bendre et al., 2017; Noguchi et al., 2018; Watanabe et al., 2021). One post-transcriptional regulation of Fam3C expression is through the RNA-binding protein poly-C binding protein 1 (PCBP1), which is phosphorylated and removed from Fam3C transcripts in response to transforming growth factor-beta (TGF-beta) signaling, allowing for Fam3C protein translation and accumulation (Hussey et al., 2011, 2012). During synthesis, Fam3C is processed through the secretory pathway, excreted from the cell, and found in extracellular vesicles (Liu et al., 2016; Thuya et al., 2023). Subcellular localization of Fam3C has been observed using immunohistochemistry (IHC) within cancerous tissue samples (Waerner et al., 2006; Csiszar et al., 2014). Localization of Fam3C to the Golgi apparatus was demonstrated by Csizar et al. (2014), who identified that the plasminogen–urokinase plasminogen activator receptor (uPAR) system is involved in processing Fam3C to its secreted form and that the cleavage and secretion of Fam3C promote tumor progression and metastasis. As a secreted protein, Fam3C can bind to the leukemia inducible factor receptor (LIFR) to enhance Stat3 activity. Fam3C extracellular signaling results in a CSC phenotype with an increased capacity for self-renewal and the increased expression of EMT and CSC factors (Woosley et al., 2019).

Here, we illustrate Fam3C’s importance in breast cancer tumor progression and metastasis using patient cohorts with TNBC. In a GEMM of metastatic cancer, conditional Fam3C knockout in mammary epithelial cells results in delayed tumor progression and decreased metastatic burden. Furthermore, Fam3C localization to the Golgi apparatus is mediated through anchoring its signal peptide into the membrane, and multiple known Golgi factors interact with Fam3C. Finally, Fam3C localization to the Golgi apparatus affects the morphology of this organelle, and consequently, alters its biological function.

## Results

### Fam3C protein levels are significantly altered in TNBC patients

**Figure 1:**
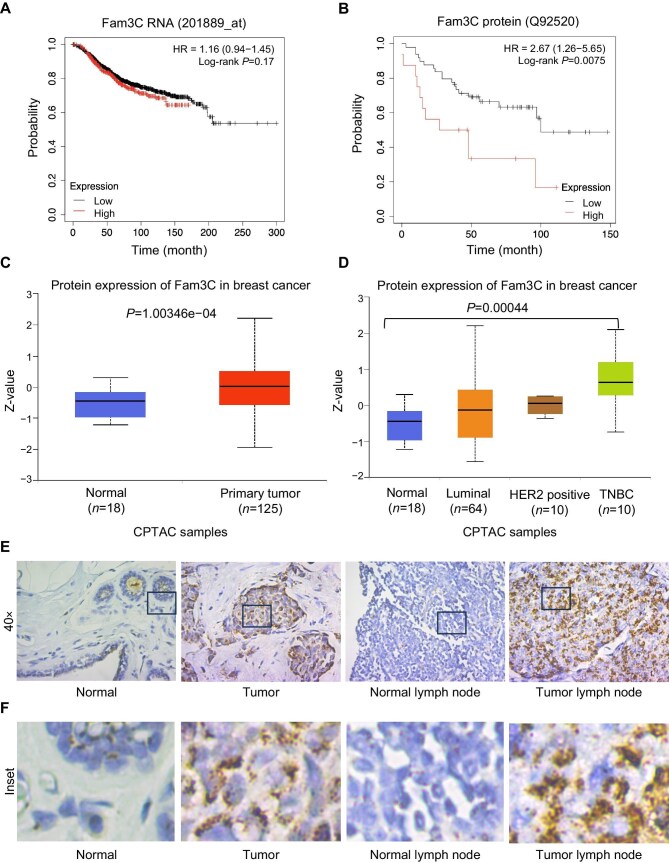
Fam3C protein levels are altered in TNBC patients. (**A** and **B**) Kaplan–Meier plots of overall survival stratifying patients in the top quartile of Fam3C mRNA expression (*P* = 0.17) (**A**) and Fam3C protein expression (*P* = 0.0075) (**B**) using KM plotter. (**C** and **D**) Protein expression of Fam3C in normal tissue vs. primary breast cancer tumor tissue (**C**) and across breast cancer major subtypes (**D**) by using UALCAN to analyze the CPTAC dataset. Additional statistical analysis can be found in Supplementary Figure S2. (**E** and **F**) Fam3C staining by IHC in tissue microarray samples across a single patient from normal mammary tissue to breast cancer tumor tissue, normal lymph node tissue, and secondary breast cancer tumor tissue within the lymph node. (**E**) 40× magnification images. (**F**) Enlargement of the inset to visualize protein localization.

Previously, our lab reported a post-transcriptional mechanism that regulates Fam3C protein expression in EMT (Hussey et al., 2011, 2012). In agreement with these findings, mRNA levels of Fam3C do not significantly correlate with overall survival in breast cancer patients. Patient samples were stratified by transcript expression of Fam3C (Figure 1A; Ősz et al., 2021), and the average overall survival period was 99 months for the low expression cohort versus 77.4 months for the high expression (upper quartile) cohort, without statistical significance. However, patients with high Fam3C protein levels (top quartile of all patient samples) displayed a significant reduction in overall survival (Figure 1B; Tang et al., 2018), with the average overall survival period of 100 months for the low Fam3C protein expression cohort versus 48 months for the high expression cohort. The distinction between mRNA abundance and protein abundance in cancer cells may contribute to previous challenges in establishing Fam3C’s relevance in cancer progression before patient protein datasets were available. The number of patient samples available in this protein dataset (Figure 1B) is still relatively low, and large-scale protein-based patient datasets in breast cancer are needed for future study.

The Clinical Proteomic Tumor Analysis Consortium (CPTAC) breast cancer dataset was analyzed using the University of Alabama at Birmingham Cancer (UALCAN) data analysis portal (Chandrashekar et al., 2017, 2022), which validated that Fam3C protein expression is significantly higher (*P* > 0.001) in primary breast tumors from patients (*n* = 125) than in normal tissues (*n* = 18) (Figure 1C; Krug et al., 2020). Among major breast cancer subtypes, TNBC samples exhibited the highest protein expression of Fam3C (Figure 1D). Additionally, within the CPTAC breast cancer cohort, samples in the highest quartile of Fam3C protein expression correlated with the highest percentage of TNBC clinical status (Supplementary Figure S1A; Cerami et al., 2012; Gao et al., 2013; Krug et al., 2020; de Bruijn et al., 2023).

Of the four subtypes of breast cancer, TNBC is more likely to metastasize and has limited therapeutic options (Yin et al., 2020). Publicly available single-cell RNA-seq data of metastatic breast cancer patients illustrate that Fam3C predominantly clusters with samples from lymph node metastases (Supplementary Figure S1B and C; Chung et al., 2017). Fam3C protein expression level also increases in lymph node metastases compared to normal lymph node cells (Woosley et al., 2019). IHC staining of Fam3C in the normal tissue adjacent to the primary tumor site, primary tumor tissue, normal lymph node, and tumor lymph node samples from one patient demonstrated higher expression of Fam3C protein in both primary tumor and tumor lymph node, as well as Fam3C localization within the cells (Figure 1E and F; Supplementary Figure S1D).

### Knockout of Fam3C in GEMMs reduces tumor burden and metastasis

**Figure 2:**
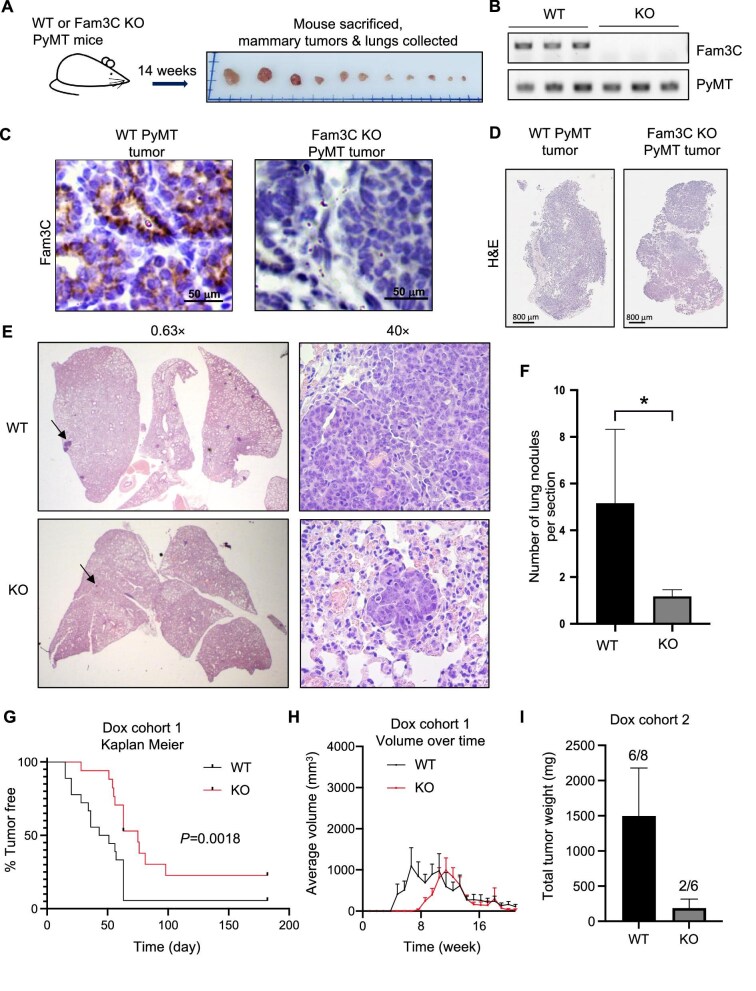
Knockout of Fam3C in engineered mouse models reduces tumor burden and metastasis. (**A**) Schematic depicting experimental procedure with WT PyMT and Fam3C KO PyMT mouse lines. Spontaneously occurring tumors were allowed to grow until 14 weeks of age when mice were sacrificed, and primary and secondary tumor sites were extracted. (**B**) Semiquantitative PCR for Fam3C and PyMT mRNA expression in WT PyMT and Fam3C KO PyMT tumor samples. (**C**) Fam3C IHC staining in WT PyMT and Fam3C KO PyMT tumors. (**D**) Representative H&E staining of WT PyMT and Fam3C KO PyMT primary mammary tumors at 1.3× magnification. (**E**) Representative images of H&E-stained lungs from WT PyMT and Fam3C KO PyMT mice at 0.63× and 40× magnification. Arrows in the 0.63× image indicate the metastatic lesion highlighted at 40×. (**F**) Quantification of lung nodules present per lung section in WT PyMT (*n* = 6) vs. Fam3C KO PyMT (*n* = 28) mice. Student’s *t*-test; *P* = 0.0125. (**G**) Kaplan–Meier plot of tumor-free percentage derived from biweekly caliper-measured volume data of Fam3C^wt/wt^;MIC;MMTV-rtTA (WT) (*n* = 18) and Fam3C^fl/fl^;MIC;MMTV-rtTA (KO) (*n* = 18) mice over time. Log-rank (Mantel–Cox); *P* = 0.0018. (**H**) Calculated volumes from biweekly caliper measurements over time of WT (*n* = 10) and KO (*n* = 12) mice. (**I**) Total primary mammary tumor weight of WT (*n* = 8) and KO (*n* = 6) mice after administration of doxycycline/sucrose water for 9 weeks.

Whole-body GEMMs have identified functional differences of Fam3C in bone morphogenesis compared to control and established that Fam3C overexpression can lead to liver fibrosis and anemia (Määttä et al., 2016; Schmidt et al., 2023). In the context of cancer progression, xenograft experiments with both murine- and human-derived cancer cell lines have demonstrated a role for Fam3C in primary tumor formation, tumor initiation, or metastatic progression depending on experiment and cancer subtype (Waerner et al., 2006; Lahsnig et al., 2009; Csiszar et al., 2014; Woosley et al., 2019; Schmidt et al., 2021). In this study, we developed a floxed Fam3C allele through Cyagen (Supplementary Figure S2A and B) to generate mice with floxed Fam3C. Then, two GEMMs of breast cancer with mammary epithelial cell-specific Fam3C knockout were used to assess primary tumor growth and metastasis. In the first model, tissue-specific Fam3C knockout was driven by Cre expression under a mouse mammary tumor virus (MMTV) promoter. At a separate genetic locus, the polyomavirus middle T (PyMT) oncogene was expressed, also under the control of the MMTV promoter. Fam3C^wt/wt^;MMTV-PyMT;MMTV-Cre (WT PyMT) and Fam3C^fl/fl^;MMTV-PyMT;MMTV-Cre (Fam3C KO PyMT) mice were allowed to age, and spontaneous mammary tumors formed with no intervention. After 14 weeks, mice were sacrificed and all primary mammary tumors from inguinal and thoracic mammary glands were extracted (Figure 2A). Primary tumor samples were weighed (Supplementary Figure S2C) and analyzed by semiquantitative polymerase chain reaction (PCR) and IHC, confirming absence of Fam3C mRNA expression (Figure 2B) and loss of Fam3C protein expression (Figure 2C) in Fam3C KO PyMT mice. Although we did not observe a significant difference in primary tumor burden between two groups of animals sacrificed at 14 weeks of age (Figure 2D; Supplementary Figure S2C), hematoxylin and eosin (H&E) staining of lung sections revealed significantly reduced lung metastases in Fam3C KO PyMT mice (Figure 2E and F). IHC staining of these lung metastases demonstrated excessive PyMT protein and Ki67 expression (Supplementary Figure S2D and E), implying that these lung lesions are in fact derived from the primary PyMT-driven mammary tumors.

In the second model, the floxed Fam3C allele was crossed with tetO-PyMT-IRES-Cre (MIC) transgene and MMTV-rtTA to drive knockout of Fam3C and PyMT expression simultaneously after administration of doxycycline through the water source. Female Fam3C^wt/wt^;MIC;MMTV-rtTA (WT) and Fam3C^fl/fl^;MIC;MMTV-rtTA (KO) mice were dispensed doxycycline/sucrose solution in their water supply at 9 weeks of age, followed by (i) continuous water administration until maximum tumor burden (2000 mm^3^) was achieved and doxycycline was removed to allow tumors to regress (Cohort 1) or (ii) continuous water administration for 9 weeks until mice were sacrificed (Cohort 2) (Supplementary Figure S2F).

In Cohort 1, Fam3C knockout significantly slowed primary tumor progression as shown by a Kaplan–Meier plot of the tumor-free percentage (Figure 2G) and volume measurements (Figure 2H) over time. In Cohort 2, 6 out of 8 WT mice bore primary tumors with an average weight of 1497.1 mg, while only 2 out of 6 Fam3C KO mice bore primary tumors with the average weight of 188 mg (Figure 2I). As expected, less surface lung nodules were observed in KO mice (1 out of 6) than in WT mice (3 out of 8) (Supplementary Figure S2H and I). These GEMMs demonstrate a role for Fam3C in the progression of spontaneous mammary tumor formation.

### Overexpression of Fam3C affects signal transduction, migration, and mammosphere formation of tumorigenic mammary cells

**Figure 3:**
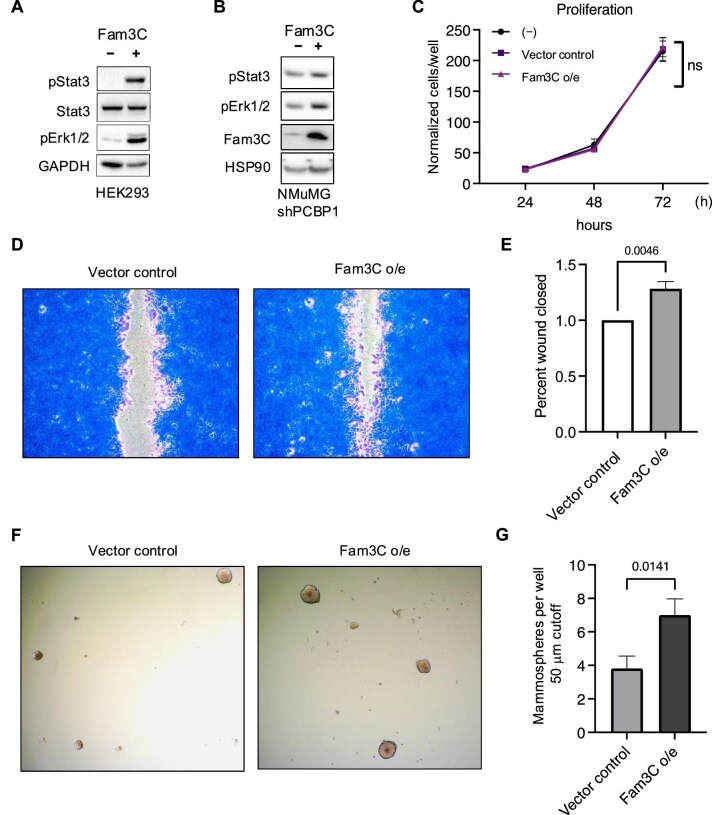
Overexpression of Fam3C initiates signaling through Stat3 and ERK to control migration and mammosphere formation. (**A**) Western blot analysis of pStat3, Stat3, pERK1/2, and GAPDH in HEK293 cells transiently overexpressing an empty vector or Fam3C. (**B**) Western blot analysis of pStat3, pERK1/2, Fam3C, and HSP90 in NMuMG shPCBP1 cells with stable overexpression of an empty vector or Fam3C. (**C**) Cell count proliferation assay at 24, 48, and 72 h in no vector control cells, empty vector control cells, and Fam3C-overexpressing NMuMG shPCBP1 cells (*n* = 3, *r* = 9; two-way analysis of variance (ANOVA); not significant). (**D**) Representative images of crystal violet-stained vector control and Fam3C-overexpressing NMuMG shPCBP1 cells 22 h after insert removal. (**E**) Quantification of wound closure assay (*n* = 4, *r* = 12; unpaired Student’s *t*-test; *P* = 0.0046). (**F**) Representative images of vector control and Fam3C-overexpressing NMuMG shPCBP1 cells grown in minimal media on ultra-low attachment plates for 10 days. (**G**) Quantification of mammosphere >50 mm in diameter (*n* = 2, *r* = 16; unpaired Student’s *t*-test; *P* = 0.0141).

To understand the effect of Fam3C modulation on breast cancer cells, we used the shPCBP1 derivative of the normal murine mammary gland (NMuMG) cell line (NMuMG shPCBP1), which was demonstrated to be tumorigenic and metastatic, as well as express and respond to Fam3C (Howley et al., 2018; Streitfeld et al., 2023). PCBP1 is a post-transcriptional regulator of Fam3C function. Upon PCBP1 knockdown, Fam3C protein expression increases. Therefore, we used the previously established Fam3C knockout derivative of the NMuMG shPCBP1 cell line (Streitfeld et al., 2023) as a clean background for our overexpression assays. Fam3C overexpression led to the activation of multiple relevant signaling cascades in tumor progression as evidenced by the phosphorylation of both Stat3 and ERK1/2 in HEK293 cells transiently overexpressing Fam3C (Figure 3A) and NMuMG shPCBP1 cells stably overexpressing Fam3C (Figure 3B). Fam3C overexpression did not alter cell proliferation (Figure 3C) but significantly increased both the migratory ability, assessed by the wound closure assay (Figure 3D and E), and the self-renewal potential, as shown by mammosphere formation (Figure 3F and G), of these cells.

### The Fam3C signal peptide anchors Fam3C protein into the Golgi membrane

**Figure 4:**
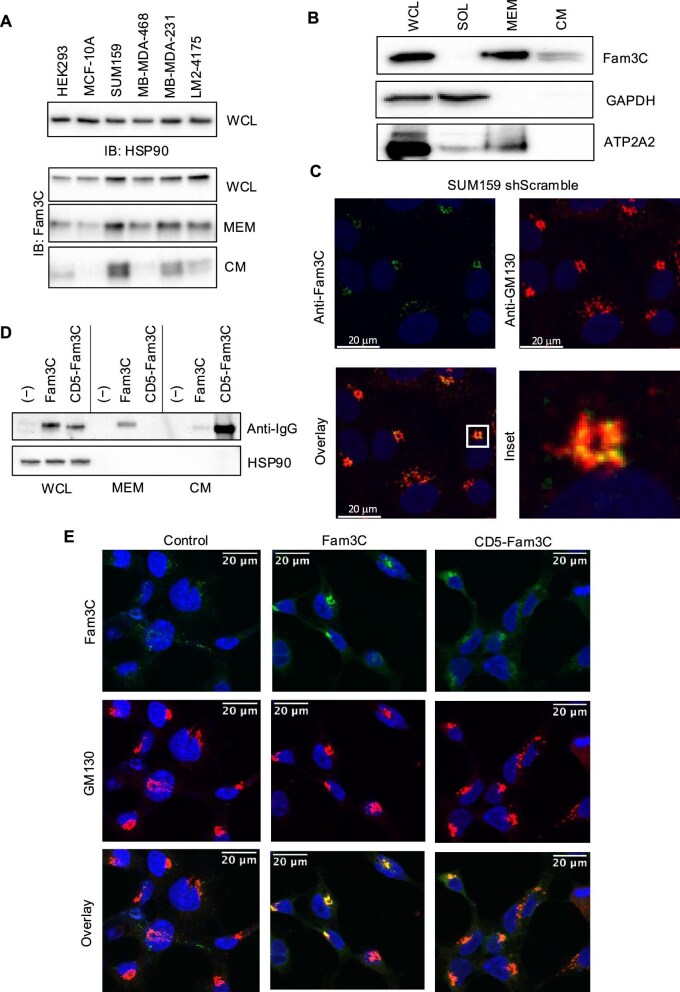
The Fam3C signal peptide anchors Fam3C protein into the Golgi membrane. (**A**) Western blot analysis of Fam3C in WCL, MEM, and CM from non-tumorigenic (HEK293 and MCF-10A) and tumor-derived (Sum159, MB-MDA-468, MB-MDA-231, and MB-MDA-231 LM2-4175) cell lines. (**B**) Western blot analysis of Fam3C in WCL, SOL, MEM, and CM from SUM159 cells. GAPDH and ATP2A2 were used as SOL and MEM markers, respectively. (**C**) Immunofluorescence staining of Fam3C (green) and GM130 (red) in SUM159 shScramble (control) cells. Overlay of the two channels and expanded inset are also depicted. (**D** and **E**) HEK293 cells were transfected with the Fam3C-Fc or CD5-Fam3C-Fc construct to overexpress wild-type Fam3C or CD5-Fam3C protein, respectively. (**D**) Western blot analysis with WCL, MEM, and CM samples from HEK293 cells overexpressing vector control, Fam3C-Fc, or CD5-Fam3C-Fc constructs. IgG antibody was used to detect the Fc-tagged proteins. (**E**) Immunofluorescence staining of Fam3C and GM130 in vector control, Fam3C-overexpressing, and CD5-Fam3C-overexpressing HEK293 cells.

Fam3C has been established as a secreted protein and also has intracellular localization (Katahira et al., 2010; Kraya et al., 2015; Liu et al., 2016). Western blot analysis for Fam3C expression in whole-cell lysates (WCL), membrane fractionation lysates (MEM), and conditioned media (CM) from a variety of human cell lines (HEK293, MCF-10A, SUM159, MB-MDA-468, MB-MDA-231, and MB-MDA-231 LM2-4157 lung metastasis derivatives) indicated higher Fam3C expression levels in breast cancer cell lines compared to normal HEK293 and MCF-10A cells in all compartments, with the highest Fam3C level in SUM159 TNBC cell line (Figure 4A). Interestingly, endogenous Fam3C protein expression was detected in the WCL, MEM, and CM from SUM159 TNBC cells, but not detected in the soluble fractions after Triton X-114 fractionation (SOL) (Figure 4B), similar to the expression patterns for Fam3C and ATP2A2 in mouse hepatocytes as observed by mass spectrometry after Triton X-114 fractionation (Mathias et al., 2011). Immunofluorescence staining also demonstrated that endogenous Fam3C protein co-localizes with the Golgi marker GM130 in SUM159 TNBC cells (Figure 4C).

Hydrophobicity plots indicated that the signal peptide of Fam3C has a higher potential to be embedded in the membrane compared to that of other secreted proteins, e.g. the purely secreted protein Ang1 (Supplementary Figure S3). Therefore, we hypothesized that the signal peptide may anchor the Fam3C protein into the membrane of secretory pathway compartments. We replaced the native signal peptide of Fam3C with that of human CD5 protein, a signal peptide commonly used to increase secretion efficiency of proteins (Zhuang et al., 2022), and found that Fam3C was no longer detected in the MEM, but only in the WCL and CM (Figure 4D), supporting that the native signal peptide anchors Fam3C in the membrane. Furthermore, immunofluorescence staining demonstrated that the CD5-Fam3C protein appears more diffuse through the cell and less co-localizing with the Golgi marker GM130, while the overexpressed wild-type Fam3C protein is contained within the more compact structures localizing with GM130 (Figure 4E). Presumably, the overall decreased signal intensity of the CD5-Fam3C protein is due to its excretion from the cell to the CM (Figure 4D).

### Fam3C localizes to the Golgi compartment and regulates Golgi morphology

**Figure 5:**
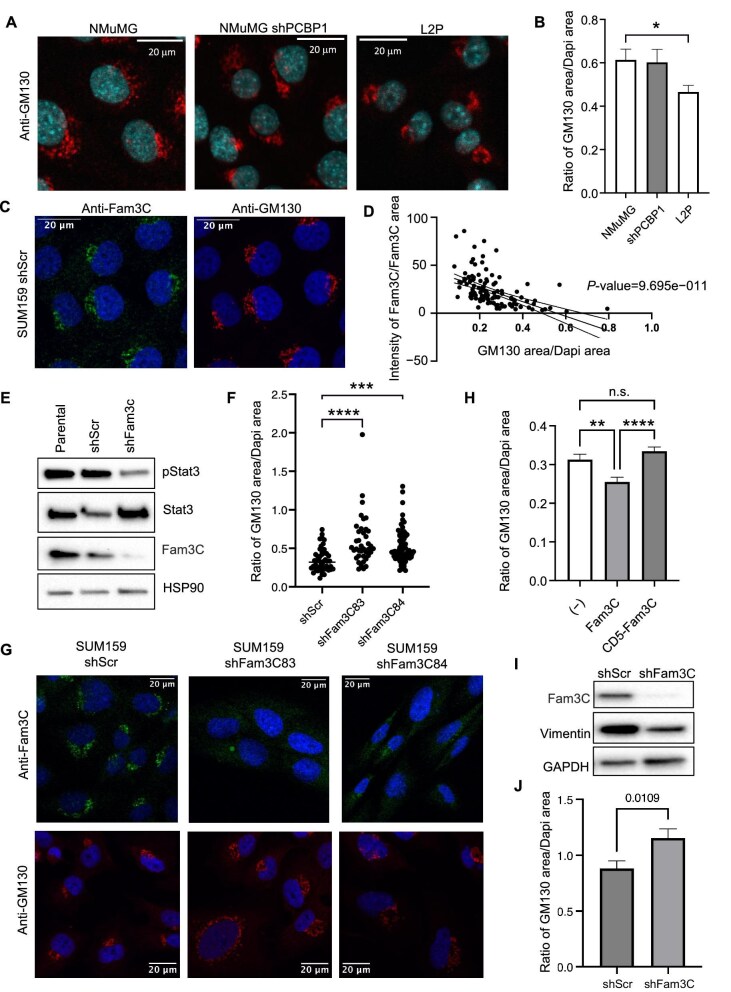
Fam3C expression alters Golgi morphology. (**A** and **B**) Immunofluorescence staining of GM130 (red) and Dapi (cyan) in NMuMG, NMuMG shPCBP1, and L2P cells. Quantification of GM130 area/Dapi area using ImageJ software (NMuMG: 37 cells; shPCBP1: 32 cells; L2P: 34 cells; *P* = 0.0159). (**C** and **D**) SUM159 shScramble (shScr) cells probed for Fam3C (green), GM130 (red), and Dapi (blue). Graphical analysis of Fam3C intensity/Fam3C area vs. GM130 area/Dapi area using ImageJ software (simple linear regression, Pearson correlation; *P* = 9.695e–11). (**E**) Western blot analysis of pStat3, Stat3, Fam3C, and HSP90 in SUM159 parental, shScr, and shFam3C cells. (**F** and **G**) SUM159 shSc, shFam3C83, and shFam3C84 cells stained for Fam3C (green), GM130 (red), and Dapi (blue). Quantification of GM130 area/Dapi area using ImageJ software (shScr: *r* = 48; shFam3C83: *r* = 39; shFam3C84: *r* = 69; one-way ANOVA multiple comparisons; shScr vs. shFam3C83 *P* < 0.0001, shScr vs. shFam3C84 *P* = 0.0002). (**H**) Quantification of GM130 area/Dapi area in HEK293 cells overexpressing vector control, Fam3C, and CD5-Fam3C using ImageJ software (*n* = 3; vector control: *r* = 141 cells; Fam3C: *r* = 141 cells; CD5-Fam3C: *r* = 190 cells; one-way ANOVA multiple comparisons; vector control vs. Fam3C *P* = 0.0059, Fam3C vs. CD5-Fam3C *P* < 0.0001, vector control vs. Fam3C not significant). (**I**) Western blot analysis of Fam3C, vimentin, and GAPDH in HMLE mesenchymal shScr and shFam3C cells. (**J**) Quantification of GM130 area/Dapi area in HMLE mesenchymal shScr and shFam3C cells using ImageJ software (shScr: *r* = 61; shFam3C: *r* = 51; *n* = 2; Student’s *t*-test, *P* = 0.0109).

Previous literature has linked Golgi morphological changes to metastatic progression of breast cancer cells (Tan et al., 2017; Marjanović et al., 2023). Knockdown of PCBP1, a key post-transcriptional regulator of Fam3C, renders the non-tumorigenic NMuMG cells tumorigenic and metastatic when introduced into non-obese diabetic–severe combined immunodeficiency (NOD–SCID) mice. The L2P stable cell line was derived by puromycin selection in 2D culture of isolated lung metastases after two rounds of NOD–SCID mammary gland xenograft injections (Howley et al., 2018) and thus is more metastatic compared to either the NMuMG or the NMuMG shPCBP1 cell line. To determine Golgi apparatus size in cells, the area of GM130 staining was measured in ImageJ and compared to the area of 4',6-diamidino-2-phenylindole (Dapi) staining per cell. Metastatic L2P cells showed statistically more compact Golgi morphology compared to the NMuMG and NMuMG shPCBP1 cell lines (Figure 5A and B).

In human SUM159 cells, we quantified the intensity of Fam3C expression/Fam3C area ratio versus Golgi area/Dapi area ratio per cell across five independent experiments (*n* = 136 cells). Fam3C intensity was significantly inversely correlated with GM130 staining area (Figure 5C and D). Then, SUM159 TNBC cells were treated with shRNA containing lentiviral particles to achieve stable introduction of a scramble sequence (shScr) or two variations of shRNA targeting Fam3C (shFam3C83 and shFam3C84). Upon effective knockdown of Fam3C expression, Stat3 phosphorylation decreased markedly (Figure 5E), the Golgi area/Dapi area ratio increased significantly (Figure 5F and G), and the fragmentation of the Golgi also increased significantly (Supplementary Figure S4A and B).

Similar changes to Golgi compaction were also observed in MDA-MB-231 cells upon stable knockdown of Fam3C (Supplementary Figure S4C–E). When SUM159 (Supplementary Figure S4F) and MDA-MB-231 (Supplementary Figure S4G) cells were treated with brefeldin A, which disrupts the endoplasmic reticulum (ER)–Golgi transport, Fam3C staining appeared to disperse and was no longer observed in distinct puncta reminiscent of Golgi morphology.

Quantification of the GM130 area/Dapi area ratio in HEK293 cells overexpressing vector control, Fam3C, and CD5-Fam3C (Figure 4E) revealed that CD5-Fam3C overexpression did not alter the overall area of the Golgi compartment, while Fam3C overexpression led to significantly reduced Golgi apparatus size (Figure 5H). Therefore, we speculate that the signal peptide of Fam3C anchors the Fam3C protein into the membrane of the Golgi apparatus, where Fam3C affects the overall morphology of the Golgi compartment.

Finally, we analyzed the mesenchymal pool of human mammary epithelial (HMLE) cells with or without Fam3C knockdown. Upon Fam3C knockdown, HMLE mesenchymal cells lose vimentin expression (Figure 5I) and increase their Golgi apparatus area simultaneously (Figure 5J; Supplementary Figure S4H), further correlating the EMT process with Golgi apparatus morphology.

### Fam3C interacts with Golgi/ER proteins and affects protein secretion and invasive potential of TNBC cells

**Figure 6:**
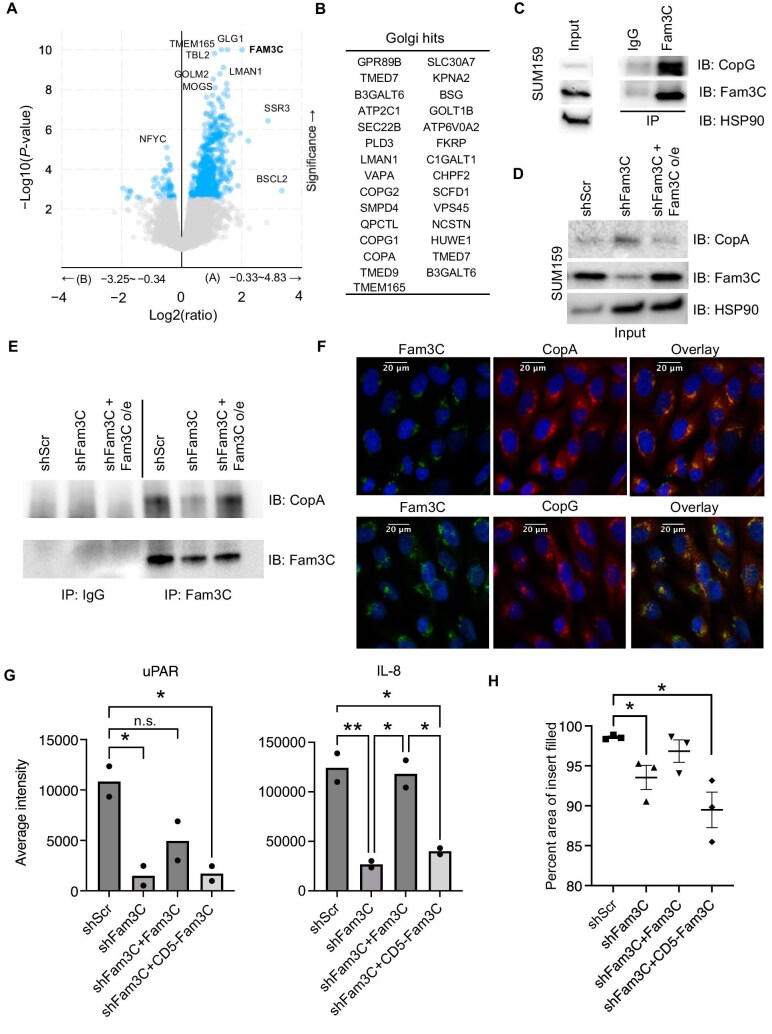
Fam3C interacts with Golgi/ER proteins and regulates protein secretion and invasion of TNBC cells. (**A**) CPTAC dataset analysis of co-regulated proteins in the highest protein abundance in breast cancer patient samples binned on median Fam3C protein abundance. (**B**) Golgi-related Fam3C interactors identified by mass spectrometry analysis of Fc-tagged Fam3C immunoprecipitation and/or Fam3C BioID proximity ligation. (**C**–**E**) SUM159 cell lysates (input) and the immunoprecipitates by IgG control or Fam3C antibody subjected to Immunoblotting with anti-CopG or anti-CopA, anti-Fam3C, and anti-HSP90 antibodies. (**F**) SUM159 cells stained for Fam3C (green), CopA or CopG (red), and Dapi (blue). (**G**) Quantification of averaged cytokine array probe intensities for uPAR and IL-8 proteins in the CM of SUM159 derivative cell lines (*n* = 2, one-way ANOVA multiple comparisons, *P < 0.05, **P < 0.001. (**H**) Quantification of the percentage of insert-filled area by SUM159 derivative cell lines in the 2D invasion assay (*n* = 3, Student’s *t*-test, *P < 0.05).

Next, we investigated potential interactors of Fam3C in the Golgi apparatus to further understand the functional role of Golgi-localized Fam3C. The analysis of proteogenomic data from CPTAC (Krug et al., 2020) highlights that Fam3C protein abundance significantly correlates with pStat3 levels (Supplementary Figure S5A and B) as well as the interaction with many Golgi apparatus and ER functional proteins. For this analysis, Fam3C was binned based on median Fam3C protein abundance. Figure 6A depicts protein abundance versus −log10(*P*-value) to demonstrate proteins in significantly higher abundance when Fam3C expression is above (positive log2(ratio) range) or below (negative log2(ratio) range) the median value in breast cancer patients, including the most significant protein GLG1 (Supplementary Figure S5C). Gene Ontology analysis for biological process enrichment revealed that these proteins significantly correlated with Fam3C expression largely function within the secretory pathways, including Golgi vesicle transport, endoplasmic reticulum to Golgi vesicle-mediated transport, protein transport, ERAD pathway, etc. (Supplementary Figure S5D).

To investigate the interaction between Fam3C and Golgi-resident proteins in cell models, C-terminal Fc-tagged Fam3C protein was immunoprecipitated from HEK293 cells and subjected to mass spectrometry analysis. A total of 109 proteins were identified as interactors by mass spectrometry analysis, among which 44 proteins were also identified as Fam3C significantly correlated proteins by the CPTAC screen (Supplementary Figure S5E). Several Golgi compartment proteins were identified and pursued for validation (Figure 6B). Notably, two members of the coatomer protein complex CopG and CopA could co-immunoprecipitate with Fam3C in SUM159 derivative cell lines (Figure 6C–E) and were found to co-localize with Fam3C by immunofluorescence staining in SUM159 cells (Figure 6F). Golgi morphology changes have been demonstrated to affect cancer cell secretome, cell migration, invasion, and metastasis (Tan et al., 2017; Marjanović et al., 2023). In addition to SUM159 shScr and SUM159 shFam3C cell lines, we also generated SUM159 derivative cell line with stable overexpression of wild-type Fam3C or CD5-Fam3C within the Fam3C knockdown background. Then, we assessed the secretory profiles of these SUM159 derivative cell lines using a cytokine array (Supplementary Figure S5F) and found that the secreted levels of uPAR and IL-8 were reduced in the CM after Fam3C knockdown, while overexpression of wild-type Fam3C, but not CD5-Fam3C, partially rescued the effect (Figure 6G). Finally, we performed the 2D invasion assay with SUM159 derivative cell lines as an initial representation of the TNBC cell capacity to invade the surrounding extracellular matrix. Fam3C knockdown significantly reduced the invasive potential of SUM159 cells, which was partially rescued by overexpression of wild-type Fam3C, but not CD5-Fam3C (Figure 6H; Supplementary Figure S5G). These results demonstrate that Fam3C localized to the Golgi compartment affects protein secretion and invasive potential of TNBC cells.

## Discussion

In this study, we demonstrate that Fam3C affects tumor progression and metastasis using conditional knockout of Fam3C in a GEMM of metastatic breast cancer. Additionally, we reveal changes in Golgi morphology, cytokine secretion, and invasion of cells when Fam3C protein is retained within the Golgi apparatus, which could be linked to metastatic potential. Fam3C interacts with multiple Golgi compartment proteins, including members of the coatomer complex, to potentially facilitate these biological functions. An area we hope to pursue in the future is to determine the mechanistic details of these protein–protein interactions that lead to altered biological functions. In addition to direct interaction with Fam3C, the expression of many Golgi compartment proteins appears to be significantly correlated with Fam3C protein abundance in breast cancer patient samples, further supporting Fam3C’s important role in this organelle. Finally, protein abundance studies in breast cancer patient cohorts reveal that Fam3C expression increases in tumor tissue compared to normal matched tissue and Fam3C protein expression correlates with triple negative status. These data indicate that Fam3C may be a valuable therapeutic target for a clinically vulnerable patient population.

Historically, Fam3C was recognized as a secreted protein, though it is indeed present in the CM of cells and the purification yield of Fam3C from the CM is low. Observing Fam3C in the Golgi apparatus is not surprising for a protein that is released into the extracellular space. However, it has not been recognized to date that Fam3C is embedded in the membrane and held within the Golgi apparatus. The effect of Fam3C on Golgi morphology and a functional role for Golgi-localized Fam3C could help distinguish between various Fam3C effects that have been observed in the field. We previously identified that Fam3C modulates CSC-related signaling events and cell outcomes through an interaction with LIFR in the extracellular space (Woosley et al., 2019). In addition, multiple groups examined Fam3C intracellular localization through IHC analysis and determined that Fam3C localization, granular versus cytoplasmic, could influence metastasis-free survival and overall survival (Waerner et al., 2006; Csiszar et al., 2014). Ciszar et al. (2014) speculated that granular localization of Fam3C represents tubular Golgi and trans Golgi network structures whereas cytoplasmic localization reflectes secretory vesicles resulting in the increased secretion of Fam3C from the cell. Analysis of secreted protein abundance in tissue samples is challenging and further investigation is needed to accurately correlate Fam3C staining with outcomes. This analysis is also complicated by necessary considerations for the plasticity of a cell undergoing metastasis. Analyzing the epithelial versus mesenchymal cell status at primary and secondary sites of tumor expansion may be valuable in future studies to understand the connection between Fam3C distribution and outcomes.

The impact of Golgi morphology on tumor progression and metastasis has been linked to EMT status (Tan et al., 2017). In our model for EMT, tumorigenesis, and metastasis (Howley et al., 2018), the metastatic line (L2P) showed a significant compaction of GM130 staining compared to the non-tumorigenic parent cells (NMuMG). Similarly, another group independently found the Golgi apparatus of post-EMT HMLE cells to be more compact than that of non-EMT control cells. Interestingly, the mesenchymal cells with compact Golgi structures were particularly susceptible to the treatment with salinomycin, which was identified to be a Golgi-disturbing agent (Marjanović et al., 2023). This is a valuable therapeutic opportunity to target cells with increased metastatic potential.

Mechanistically, the retention of Fam3C within the Golgi apparatus is likely regulated through protein–protein interactions localized to that compartment. We discovered multiple potential Fam3C-interacting proteins that are known to have Golgi-related functions. Several members of the coatomer complex, including CopG1 and CopA, were identified and validated in SUM159 TNBC cells. The coatomer complex is typically involved in protein trafficking from the Golgi compartment to the ER but is also capable of trafficking between Golgi compartments. High expression of coatomer complex proteins is associated with poor prognosis in ovarian and breast cancer (Claerhout et al., 2012). CopA has been determined to correlate with poor overall survival in cervical cancer as well (Bao et al., 2022). Furthermore, knockdown of CopA in MDA-MB-231, MDA-MB-468, and PC3 cells resulted in decreased cell viability through apoptosis (Shtutman et al., 2011; Claerhout et al., 2012). PC3 cells specifically displayed fragmentation and disappearance of the Golgi apparatus upon CopA silencing (Shtutman et al., 2011). The coatomer complex could help maintain Fam3C localization. Future investigation should identify a mechanistic role for the interaction between Fam3C and the coatomer components in the pathogenesis of Golgi structure and function.

As previously discussed, Fam3C is known to be excreted from the cell. uPAR has been identified as a key player in the processing and release of Fam3C (Csiszar et al., 2014). Interestingly, uPAR was one of the regulated proteins within our cytokine array affected by Fam3C expression. This could be a feedback mechanism that ultimately regulates Fam3C excretion. The biological switch that results in Fam3C release from the Golgi apparatus into the extracellular space is a key missing component in our understanding of Fam3C physiological function, particularly the Fam3C biological activity within each compartment. Localization within the Golgi apparatus affects Golgi morphology, protein secretion, and invasion, whereas secreted Fam3C signaling events have been linked to stemness properties and tumor initiation. Further investigation into the biological context for the release of Fam3C from the cell will be a valuable component to explain its role in tumor progression.

## Materials and methods

Comprehensive materials and methods can be found in Supplementary material.

### Animal models

All animal procedures were approved by the Animal Care and Use Committee of the Medical University of South Carolina. The floxed Fam3C mouse model generated by Cyagen utilized LoxP sites flanking exons 4 and 5 of the Fam3C gene (Supplementary Figure S2A) and was crossed with two murine breast cancer models, (i) MMTV-PyMT;MMTV-Cre mice and (ii) MMTV-rtTA;MIC mice, respectively, in an FVB background. Fam3C^fl/fl^ or Fam3C^wt/wt^ mice crossed with MMTV-rtTA;MIC mice (Rao et al., 2014) were administered doxycycline solution (2 mg/ml doxycycline hyclate, Sigma, #D9891-25G, with 10 mg/ml sucrose, Sigma, #S9378-500G) within the water supply at 9 weeks of age. For Cohort 1, the doxycycline/sucrose water was continuously administered until a tumor burden of 2000 mm^3^ was reached, after which tumors were allowed to regress. For Cohort 2, mice were administered doxycycline/sucrose water for 9 weeks and sacrificed to access tumor burden at 18 weeks of age.

### Immunofluorescence

Cells were plated onto poly-L-lysine-coated coverslips in their normal media. After 24–48 h, cells were fixed in 4% paraformaldehyde before blocking and incubation with appropriate primary and secondary antibodies. Images were processed and overlayed, and areas and intensities were measured using ImageJ software. Fractionation was determined by thresholding GM130 images and particle analysis in ImageJ software.

### Membrane fractionation

Cells were washed in phosphate-buffered saline and lysed in 1× Triton X-114 buffer for 30 min on ice. Samples were spun at 10000× *g* for 5 min at 4°C to remove nuclei. Supernatants were transferred to a fresh tube and warmed to 32°C for 3 min and quickly centrifuged at 10000× *g* for 20 sec at room temperature. Aqueous and hydrophobic phases were reextracted, and samples were prepared for immunoblotting.

## Supplementary Material

mjaf042_Supplemental_File

## Notes

**Author contributions:** A.C.D. conceived and performed experiments, analyzed data, and wrote the manuscript. E.R.M.R. and B.V.H. performed experiments and assisted with animal colony generation and maintenance. W.S.S. and C.F. performed specialized techniques. P.H.H. conceived experiments and supervised the study, and P.H.H. and B.V.H. provided guidance and edited the manuscript. Funding was acquired by A.C.D., B.V.H., and P.H.H.
