# Supplementary material for: Fam3C alters Golgi apparatus morphology and function in triple negative breast cancer
Source: J Mol Cell Biol. 2025 Nov 17;17(11):mjaf042. doi: 10.1093/jmcb/mjaf042 (PMC13198017; doi:10.1093/jmcb/mjaf042)
Supplement: mjaf042_Supplemental_File [file mjaf042_supplemental_file.pdf]

Supplementary Figure S1. Fam3C protein levels are altered in triple negative breast cancer patients. Related to Figure 1.

A

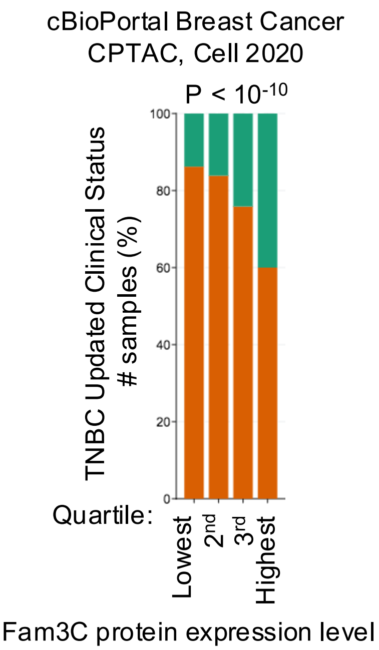

B

Comparison	Statistical significance
**Normal-vs-Luminal**	**4.088723E-03**
Normal-vs-HER2 Positive	3.780225E-01
**Normal-vs-TNBC**	**4.403405E-04**
Luminal-vs-HER2 Positive	2.999434E-01
**Luminal-vs-TNBC**	**4.215500E-02**
**HER2 Positive-vs-TNBC**	**1.548081E-02**

C

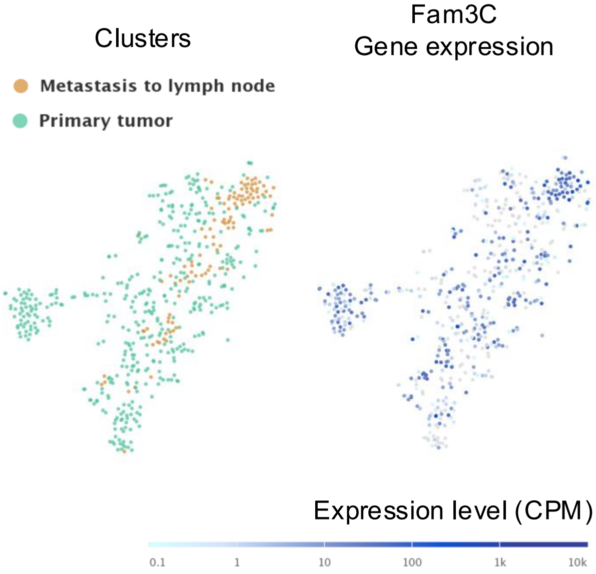

D

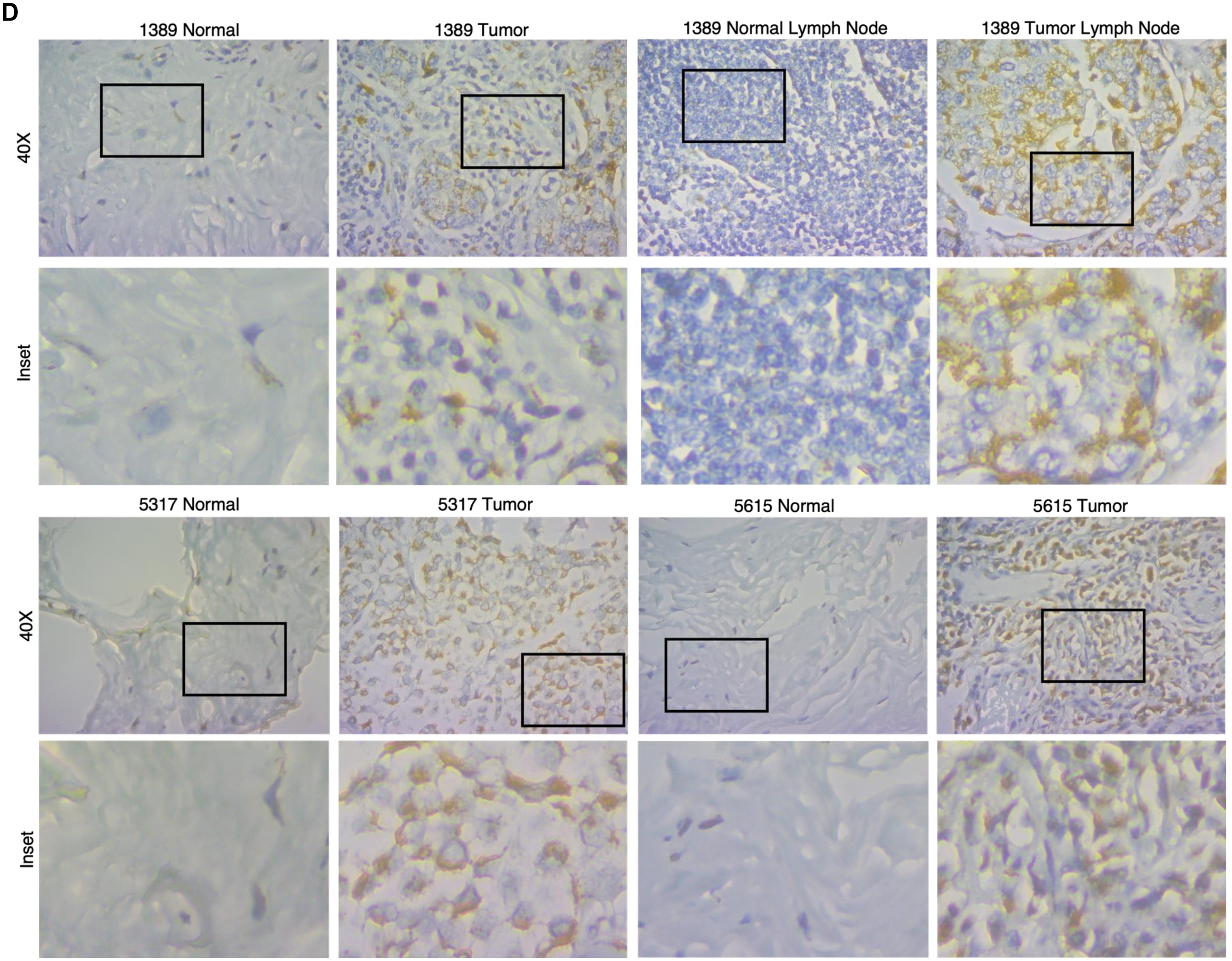

Supplementary Figure S1. Fam3C protein levels are altered in triple negative breast cancer patients. Related to Figure 1.

(A) cBioportal analysis of TNBC status in patient samples binned by Fam3C protein abundance ($p<10^{-10}$). (B) Complete statistical analysis of data shown in Figure 1D from UALCAN analysis of the CPTAC proteomic breast cancer dataset. (C) Single Cell Expression Atlas EMBL-EBI analysis of single cell RNA-seq data from Chung W., et al. using cells isolated from 11 breast cancer patients. (D) Additional patient Fam3C staining by IHC in tissue microarray samples from normal mammary tissue to breast cancer tumor tissue, normal lymph node tissue and secondary breast cancer tumor tissue within the lymph node; 40× magnification images, numbers represent de-identified samples.

Supplementary Figure S2. Knock out of Fam3C in engineered mouse models reduces tumor burden and metastasis. Related to Figure 2.

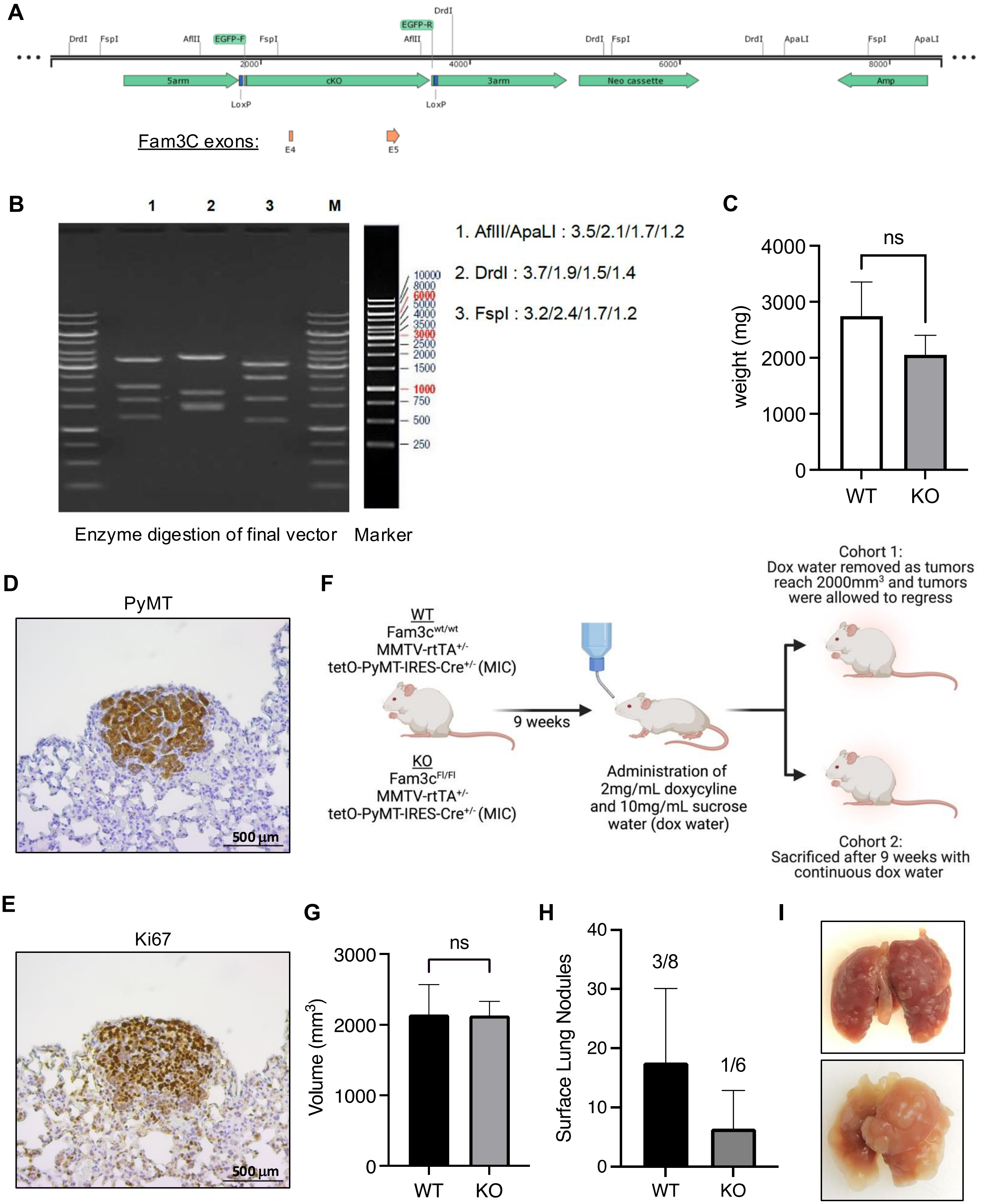

Supplementary Figure S2. Knock out of Fam3C in engineered mouse models reduces tumor burden and metastasis. Related to Figure 2.

(A) Vector schematic designed by Cyagen for targeted excision of exons 4 and 5 of the Fam3C gene. (B) Restriction enzyme digestion validation of targeting vector completed by Cyagen. (C) Total primary tumor weights of age matched WT (Fam3C$^{wt/wt}$; MMTV-PyMT; MMTV-Cre) and KO ((Fam3C$^{fl/fl}$; MMTV-PyMT; MMTV-Cre) mice. (WT: n=10, KO: n=30; Statistical test: Student's t-test. Not significant). (D) IHC analysis of PyMT and (E) Ki67 protein in a formalin-fixed paraffin embedded lung tissue section from a WT (Fam3C$^{wt/wt}$; MMTV-PyMT; MMTV-Cre) mouse. (F) Schematic representing the 2 cohorts examined experimentally using the MIC mouse model; WT (Fam3C$^{wt/wt}$; MIC; MMTV-rtTA) or KO (Fam3C$^{fl/fl}$; MIC; MMTV-rtTA) mice. Figure generated using Biorender.com. (G) Average volume of tumors measured by digital calipers at the time of Dox water removal for Cohort 1 using the MIC mouse model. (WT: n=10, KO: n=12; Statistical test: Student's t-test. Not significant). (H) Quantification of surface lung nodules for Fam3C$^{wt/wt}$ (n=8) or Fam3C$^{fl/fl}$ (n=6); MIC; MMTV-rtTA mice. (I) Lung images of samples with the highest metastatic tumor burden for each of the Fam3C$^{wt/wt}$ and Fam3C$^{fl/fl}$; MIC; MMTV-rtTA groups.

Supplementary Figure S3. The Fam3C signal peptide anchors Fam3C protein into the Golgi membrane. Related to Figure 4.

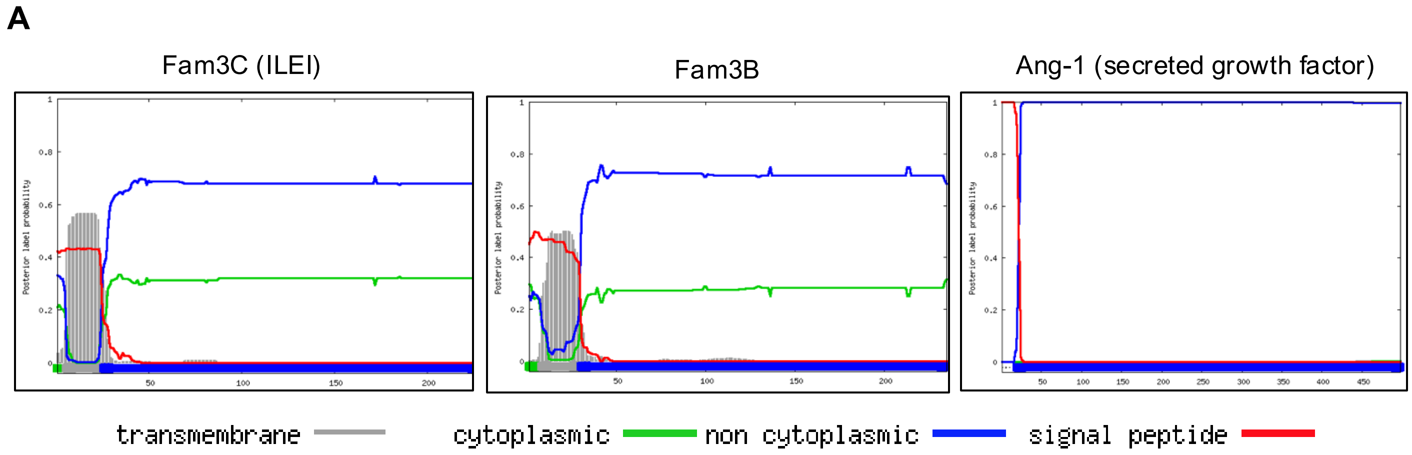

Supplementary Figure S3. The Fam3C signal peptide anchors Fam3C protein into the Golgi membrane. Related to Figure 4.

(A) Polyphobius hydrophobicity plots and predictions for Fam3C, Fam3B and Ang-1.

Supplementary Figure S4. Fam3C expression alters Golgi morphology. Related to Figure 5.

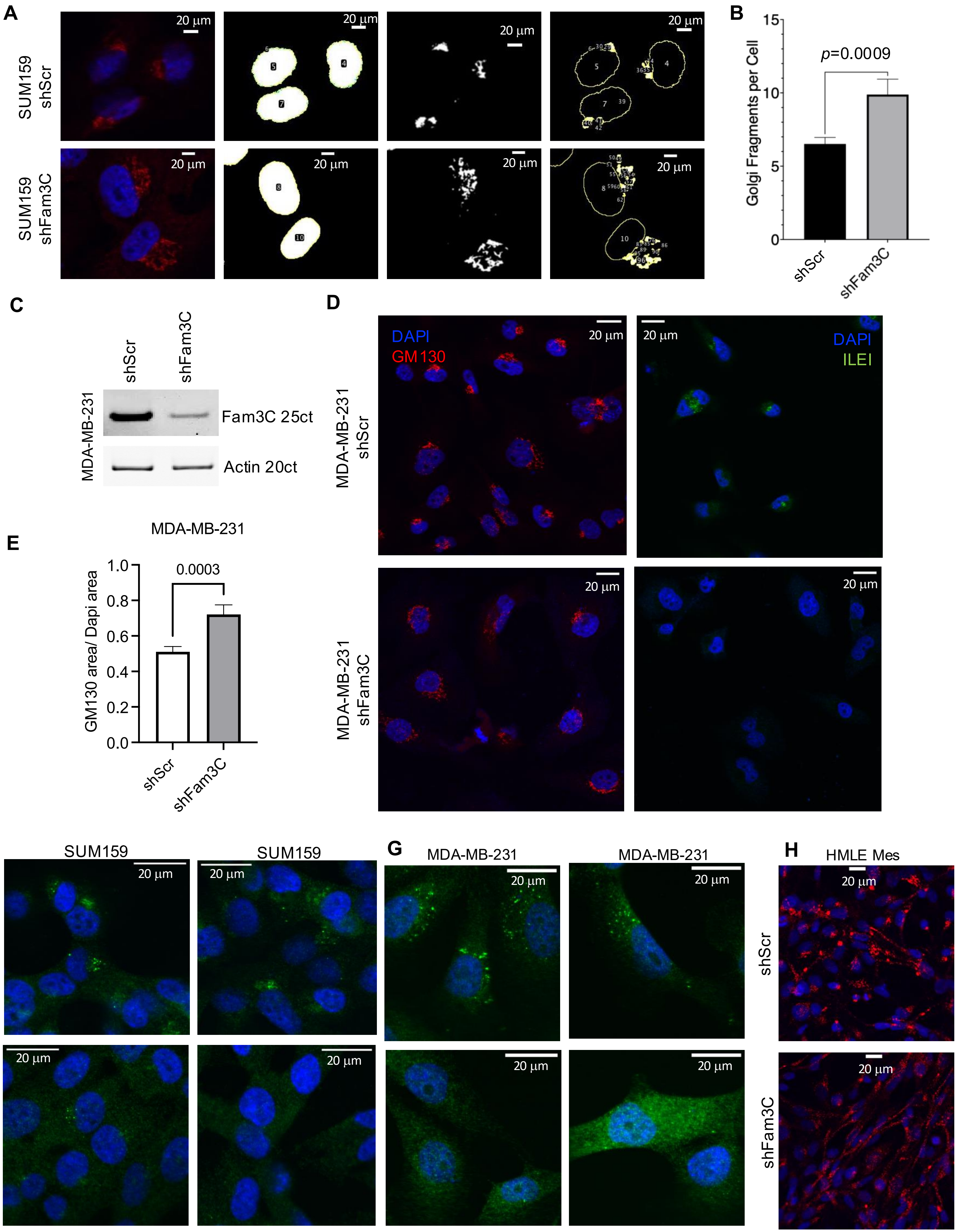

Supplementary Figure S4. Fam3C expression alters Golgi morphology. Related to Figure 5.

(A) Representative images from GM130 and Dapi stained SUM159 shScramble and shFam3C cells thresholded in ImageJ and subjected to particle analysis to determine fractionation of the Golgi apparatus. (B) Quantification of Golgi fragments per cell (r=116 cells (shScramble) and 70 cells (shFam3C); n=2; Student's t-test, p=0.0009). (C) Semiquantitative PCR using Fam3C (25 PCR cycles) and Actin (20 PCR cycles) targeted primer sets to assess mRNA abundance in MDA-MB-231 lentiviral mediated control (shScramble) or Fam3C knockdown (shFam3C) cells. (D) Representative immunofluorescence images of MDA-MB-231 shScramble and ShFam3C cells probed for Fam3C (green), GM130 (red) and Dapi (blue). (E) Quantification of GM130 area/Dapi area measured using ImageJ software (r= 137 (shScramble) and 82 cells (shFam3C); n= 2) Statistical test: Student's t test, p=0.0003). (F) Two representative immunofluorescence images each of vehicle control or brefeldin A (BFA) treated SUM159 cells probed for Fam3C (green) and Dapi (blue). (G) Two representative immunofluorescence images each of vehicle control or brefeldin A (BFA) treated MDA-MB-231 cells probed for Fam3C (green) and Dapi (blue). (H) Representative images of HMLE mesenchymal shScramble and shFam3C stable cell pools; Fam3C (green), Dapi (blue) and GM130 (red).

Supplementary Figure S5. Fam3C interacts with Golgi/ER resident proteins and regulates protein secretion and invasion. Related to Figure 6.

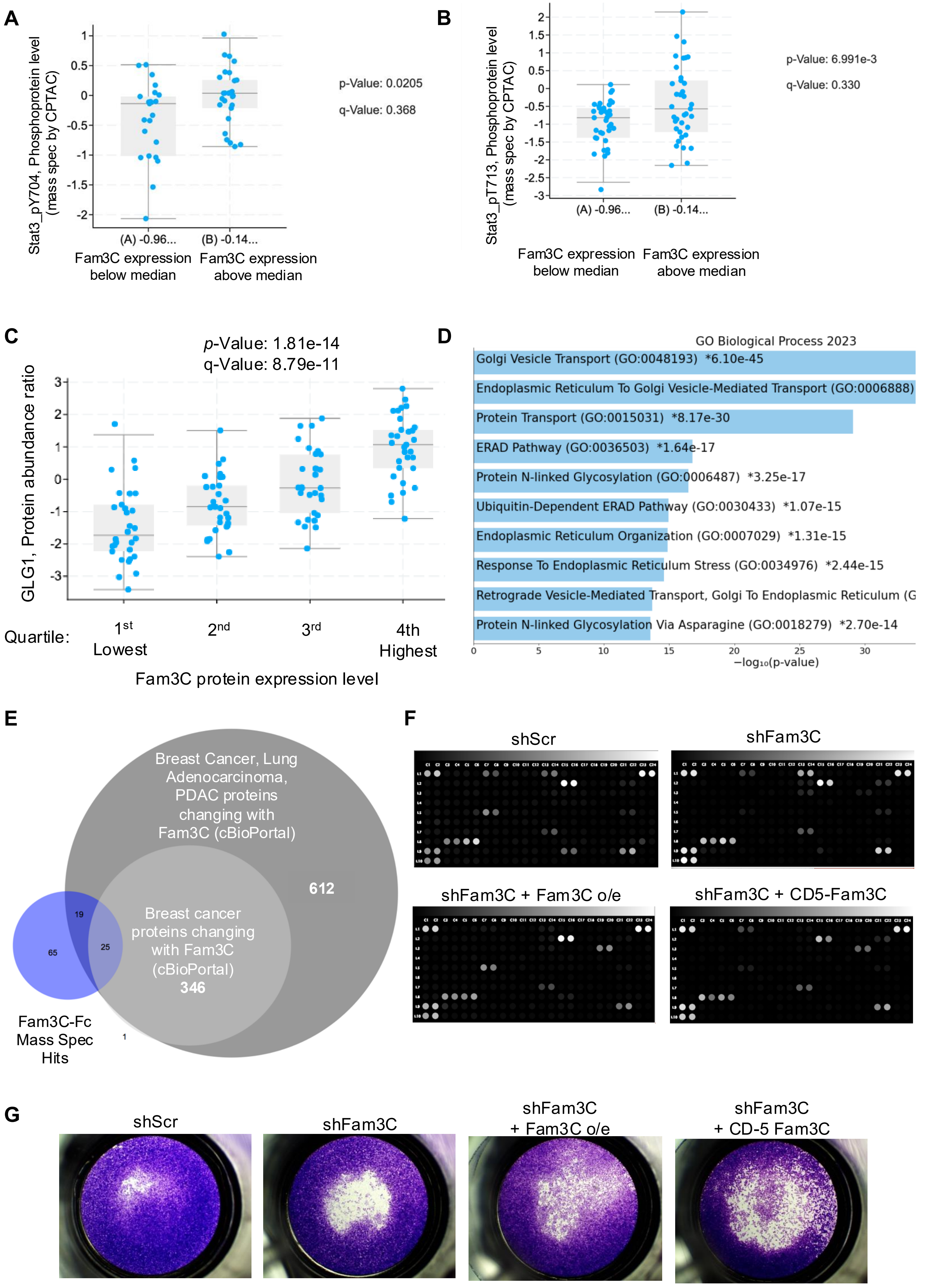

Supplementary Figure S5. Fam3C interacts with Golgi/ER resident proteins and regulates protein secretion and invasion. Related to Figure 6.

(A) cBioPortal analysis of phosphoStat3_pY704 phosphoprotein site level expression in human CPTAC patient samples binned by median Fam3C protein abundance (*p*=0.0205). (B) cBioPortal analysis of phosphoS3_pT713 phosphoprotein site level expression in human CPTAC patient samples binned by median Fam3C protein abundance (*p*=6.991e-3). (C) cBioPortal analysis of GLG1 protein abundance in human CPTAC patient samples binned by quartiles based on Fam3C protein abundance. (*p*=1.81e-14). (D) Enrichr analysis of Fam3C significantly correlated proteins from the CPTAC dataset using the Go Biological processes reference list. (E) BioVenn overlap of genes identified through Fam3C-Fc IP mass spectrometry compared to the cBioportal CPTAC breast cancer Fam3C correlated protein list and a cBioPortal list of proteins significantly correlated with Fam3C in Breast Cancer, Lung Adenocarcinoma and PDAC combined. (F) Spot grids from the Bio-Techne Proteome Profiler Human Cytokine Array Kit incubated with conditioned media from SUM159 shScramble, shFam3C, shFam3C + Fam3C o/e and shFam3C + CD5-Fam3C o/e cells. (G) Representative images of crystal violet stained 2d invasion inserts incubated with SUM159 shScramble, shFam3C, shFam3C + Fam3C o/e and shFam3C + CD5-Fam3C o/e cells.

Supplementary Table S1.

Primer	Sequence (5' → 3')	Use
shScramble (shSCR)	CCGGTCCTAAGGTTAAGTCGCCCTCGCTCGAGCGAGGGCGA CTTAACCTTAGGTTTTTG	Lentiviral shRNAs
shFam3C83 (TRCN0000298583)	CCGGATGTTGGAAGAGGGATCAATGCTCGAGCATTGATCCCT CTTCCAACATTTTTTG	Lentiviral shRNAs
shFam3C84 (TRCN0000298584)	CCGGGAGGAGATGTGGCACCATTTACTCGAGTAAATGGTGC CACATCTCCTCTTTTTG	Lentiviral shRNAs
Cre 320nt For	GAA CCT GAT GGA CAT GTT CAG G	Genotyping
Cre 320nt Rev	AGT GCG TTC GAA CGC TAG AGC CTG T	Genotyping
Cre 200nt For	ATT GCT GTC ACT TGG TCG TGG C	Genotyping
Cre 200nt Rev	GGA AAA TGC TTC TGT CCG TTT GC	Genotyping
PyMT A For	AGC CCG ATG ACA GCA TAT CC	Genotyping
PyMT A Rev	GGT CTT GGT CGC TTT CTG GAT	Genotyping
PyMT B For	AGT CAC TGC TAC TGC ACC CAG	Genotyping
PyMT B Rev	CTC TCC TCA GTT CTT CGC TCC	Genotyping
PyMT C For	TCC AAC AGA TAC ACC CGC AC	Genotyping
PyMT C Rev	ATG AGC CCT CTG CAA ATC CC	Genotyping
Fam3C 5' LoxP site For	GAG AAA TTC AGG TTC CCC AAC	Genotyping
Fam3C 5' LoxP site Rev	AAA TGA TGC TGC TTC TGC TG	Genotyping
Fam3C 3' LoxP site For	TCC CCT GTG ACA AAG AGA CC	Genotyping
Fam3C 3' LoxP site Rev	CAT GCA TTG CTC TGG GTA GA	Genotyping
CD5 Signal Peptide in pFuse-hIgG2-Fc1 BamH1/ Kpn1 Forward	CCGGTATGCCCATGGGGTCTCTGCAACCGCTGGCCACCTTG TACCTGCTGGGGATGCTGGTCGCTTCCTGCCTCGGAGGTAC	Cloning
CD5 Signal Peptide in pFuse-hIgG2-Fc1 BamH1/ Kpn1 Reverse	CTCCGAGGCAGGAAGCGACCAGCATCCCCAGCAGGTACAAG GTGGCCAGCGGTTGCAGAGACCCCATGGGCATA	Cloning
CD5 Signal Peptide in pcDNA 3.1+ BamH1/ Age1 Forward	GATCCATGCCCATGGGGTCTCTGCAACCGCTGGCCACCTTGT ACCTGCTGGGGATGCTGGTCGCTTCCTGCCTCGGAA	Cloning
CD5 Signal Peptide in pcDNA 3.1+ BamH1/ Age1 Reverse	CCGGTTCCGAGGCAGGAAGCGACCAGCATCCCCAGCAGGTA CAAGGTGGCCAGCGGTTGCAGAGACCCCATGGGCATG	Cloning
hILEI codon optimized target sequence with native signal peptide BamH1 Forward	ggtggtGGATCCATGAGAGTCGCTGGAGCCGCTAAAC	Cloning
hILEI codon optimized target sequence to remove signal peptide in pFuse-hIgG2-Fc1construct Kpn1 Forward	TTGGGGTACCCAGGTGTTCGAGATTAAGATGG	Cloning
hILEI codon optimized target sequence to remove signal peptide pcDNA3.1 construct Age1 Forward	TTGGACCGGTCAGGTGTTCGAGATTAAGATGG	Cloning
hILEI codon optimized target sequence Xho1 Reverse	ggtggtCTCGAGGTCTTGCTTCTGGGGGATGC	Cloning
Fam3C Forward	GAGCTGCAAAGTTGGTAGTG	Semiquant PCR
Fam3C Reverse	TAGAACGAATGGCTGAGTCC	Semiquant PCR
Actin Forward	ATG CTT CTA GGC GGA CTA TG	Semiquant PCR
Actin Reverse	ACA AAT AAA GCC ATG CCA AT	Semiquant PCR

Supplementary Table S1.
All primers used for included experimentation.

Supplementary Materials and Methods

Bioinformatic analysis of publicly available patient datasets

KM plotter (Ősz et al., 2021) was used to stratify high Fam3C expressing mRNA using the Affy ID 201889_at and protein samples using the upper quartile cutoff. Overall patient survival Kaplan-Meier plots were generated for both mRNA and protein datasets (Tang et al., 2018).

Analysis of the Clinical Proteomic Tumor Analysis Consortium (CPTAC) proteogenomic landscape of breast cancer dataset (Krug et al., 2020) was completed in part using the UALCAN website (Darshan S. Chandrashekar et al., 2017; Darshan Shimoga Chandrashekar et al., 2022). Fam3C protein expression was compared in normal versus primary breast cancer tumor samples, as well as normal versus luminal, HER2 positive and TNBC breast cancer patient samples. cBioPortal (Cerami et al., 2012; Gao et al., 2013; de Bruijn et al., 2023) was used to investigate Fam3C protein levels within the proteogenomic landscape of breast cancer (CPTAC) dataset (n=122) (Krug et al., 2020). Fam3C protein expression was binned by quartile or median as stated to evaluate TNBC status, total protein correlation, or phosphoprotein correlation. Enrichr (Chen et al., 2013) was used to categorize proteins that were significantly correlated with protein abundance using the Gene Ontology reference list GO Biological Processes (Consortium et al., 2023). Similar analysis as above comparing Fam3C protein abundance to other proteins was completed in a CPTAC lung adenocarcinoma sample dataset (Gillette et al., 2020) and CPTAC PDAC sample dataset (Cao et al., 2021) using cBioPortal. BioVenn was used to overlay the results with mass spectrometry hits (Hulsen et al., 2008). Single cell RNA-seq data was analyzed using the Single Cell Expression Atlas through EMBL-EBI and data from (Chung et al., 2017). PolyPhobius was used to predict transmembrane regions of the protein FASTA sequences for Fam3C, Fam3B, TNFalpha and Ang1 (Ott and Lingappa, 2002).

Tissue microarrays and immunohistochemistry

Blinded patient Tissue Microarray samples were processed using Fam3C antibody (Sigma HPA050548, 1:1,600) and hematoxylin counterstain for immunohistochemistry analysis as described previously (Woosley et al., 2019).

Animal Models

All animal procedures were approved by the Animal Care and Use Committee of the Medical University of South Carolina. A Fam3C floxed mouse model was generated by Cyagen. LoxP sites were introduced on either side of exons 4 and 5 of the Fam3C gene **(Supplementary Fig. 2A)**. Validation of the targeting vector is shown in **Supplementary Figure 2B**. This conditional floxed Fam3C allele was crossed with two murine breast cancer models; (1) MMTV-PyMT; MMTV-Cre mice or (2) MMTV-rtTA; tetO-PyMT-IRES-Cre (MIC) mice in an FVB background.

Fam3C$^{fl/fl}$ or Fam3C$^{wt/wt}$; MMTV-PyMT (The Jackson Laboratory; strain #003553 (Wagner et al., 1997)); MMTV-Cre (The Jackson Laboratory; strain #002374 (Lin et al., 2003)) females were sacrificed at 14 weeks of age, a time point determined to not exceed maximum tumor burden as defined in the MUSC IACUC protocol of 4000mg. At the 14-week endpoint, mice were sacrificed, and samples were collected as described below.

Alternatively, Fam3C$^{fl/fl}$ or Fam3C$^{wt/wt}$ mice were crossed with MMTV-rtTA; tetO-PyMT-IRES-Cre (Rao et al., 2014). At 9 weeks of age, doxycycline (Doxycycline hyclate, Sigma, #D9891-25G) was administered at a concentration of 2mg/mL in combination with 10mg/mL sucrose (Sigma, #S9378-500G) within the water supply. For cohort 1, the doxycycline/sucrose water was continuously administered until a tumor burden of 2000mm^3 was reached as determined by biweekly volumetric digital caliper measurements. As this tumor burden was attained for each individual mouse, doxycycline/sucrose water was replaced with normal water, and tumors were allowed to regress. Tumors were monitored until tumors regressed with digital caliper measurements. Cohort 2 was administered doxycycline/sucrose water at 9 weeks of age and

was subsequently monitored biweekly for 9 weeks with continuous doxycycline/sucrose water administration. This second cohort was sacrificed to access tumor burden at a total age of 18 weeks.

All animals were monitored for tumors and once palpable were monitored twice weekly and measured using digital calipers for volume calculations. For all cohorts, mice were sacrificed according to IACUC standards, mammary tumors and lungs were extracted, mammary tumors were weighed, partitioned and samples were either flash frozen for RNA extraction, or fixed in 10% formalin (Fisher) for 24h before storage in 70% ethanol at 4 degrees Celsius. Formalin fixed tissues were paraffin embedded, sectioned at 5μm, and analyzed by immunohistochemistry as described previously (Woosley et al., 2019) by the Biorepository and Tissue Analysis Resource at MUSC. Whole tumor and lung sections were imaged using a Leica MZ 95 microscope with an Amscope MU130 camera and Amscope acquisition software. 5x-40x images were acquired with a Leica DMIL LED microscope with an Amscope MU500 camera and Amscope acquisition software. Surface lung nodules or lung section lesions identified by hematoxylin and eosin (H&E) were counted by three blinded researchers and averaged.

Tissue disruption, Reverse Transcription and semi-quantitative PCR

Total RNA was extracted from flash-frozen tumor samples by placing them in tubes containing 1.4 mm ceramic beads (VWR, 10158-610; OMNI International, 19-627D) and homogenizing them in TRIzol reagent (Ambion) using a mini bead mill homogenizer (VWR) for four cycles of 30 seconds at maximum speed. Chloroform was then added before carrying out the rest of the extraction according to the manufacturer's instructions. cDNA synthesis was performed using qScript cDNA synthesis kits with 1μg of total RNA (Quantabio). Maxima HotStart Taq Polymerase Mix (ThermoFisher) was used for PCR reactions with 10ng cDNA. Primers used for semiquantitative PCR experiments are listed in **Supplementary Table S1**. 9% Acrylamide: Bis-acrylamide (36.5:1) (Fisher, BP170-500; Bis-Acrylamide, Fisher, BP171-100) gels were run in 1x TBE to visualize PCR products and imaged in ethidium bromide.

Cell culture

Cell lines were obtained from ATCC with the exception of SUM159 cells which were a generous gift from Dr. Ethier (Ethier et al., 2020) (Medical University of South Carolina). HMLE and LM2-4175 MDA-MB-231 derivatives which were generously gifted by Dr. Massague (Minn et al., 2005) (Sloane Kettering Institute). MB-MDA-231, LM2-4175, MDA-MB-468 and HEK293 cells were cultured in DMEM (with 4.5 g/L glucose, L-glutamine and sodium pyruvate) (Corning, #10-013-CV) supplemented with 10% Fetal Bovine Serum (FBS) (R&D systems Bio-techne, #S11150), 1:100 Antibiotic-Antimycotic (Gibco, #15240-062), 0.05mg Plasmocin prophylactic (InvivoGen, #ant-mpp). SUM159, NMuMG and NMuMG derivatives were cultured in DMEM (4.5 g/L glucose, L-glutamine and sodium pyruvate) (Corning, #10-013-CV) supplemented with 5% FBS (R&D systems Bio-techne, #S11150), 5% Bovine Calf Serum (BCS) (HyClone, #SH30073.04), 1:100 Antibiotic-Antimycotic (Gibco, #15240-062), 0.05mg Plasmocin prophylactic (InvivoGen, #ant-mpp). NMuMG shPCBP1 Fam3C Crispr Cas9 knockout cells were generated and validated as previously described (Streitfeld et al., 2023). MCF-10A cells were cultured in DMEM F12 (Gibco #11330-032) with 5% BCS (HyClone, #SH30073.04), 0.5mg/ml hydrocortisone (Sigma #H-0888), 10ug/ml insulin (Sigma #I0516-5ml), 20ng/ml EGF (Corning #354052), 100ng/ml Cholera Toxin (Sigma #C-8052), 1:100 Antibiotic-Antimycotic (Gibco, #15240-062). Cells were tested regularly for mycoplasma contamination using the MycoStrip mycoplasma detection kit (InvivoGen; rep-mys-10). HMLE cells were cultured in DMEM:F12 (Gibco, #11330-032) supplemented with 5% calf serum (BCS) (HyClone, #SH30073.04), 10ug/ml of insulin (Sigma, #I0516), 0.5ug/ml of hydrocortisone (Sigma, #H0888-1G), 20ng/ml of epidermal growth factor (Corning, #354052), 1:100 Antibiotic-Antimycotic (Gibco, #15240-062). The mesenchymal pool of HMLE cells used in this study were previously isolated using CD44/CD24 markers by flow cytometry (Woosley et al., 2019).

Cloning and generation of stable cell lines

Codon optimized Fam3C sequence (as previously described (Woosley et al., 2019)) was cloned into the pFuse-hIgG2-Fc1 vector (InvivoGen, pfuse-hfc1) with either the native signal peptide (Fam3C amino acids 1-227) or the CD5 signal peptide (MPMGSLQPLATLYLLGMLVASCLG + Fam3C amino acids 25-227). The Kpn1 restriction enzyme was used for cloning of the CD5 construct and therefore introduces two amino acids (glycine and threonine) between the CD5 signal peptide and the included Fam3C sequence. Vectors were originally introduced into cells using Lipofectamine 3000 reagent (Invitrogen, #L3000015) according to manufacturer's instructions. pFuse-hIgG2-Fc1 vectors were selected for using zeocin (Invitrogen, 46-0509; 100μg/ml) to generate stable overexpression cell pools.

Lentiviral constructs were obtained from the MUSC Hollings Cancer Center shRNA Shared Resource Technology. These Mission pLKO.1-puromycin constructs (hairpin sequences are listed in **Supplementary Table S1**) were introduced into HEK293t cells with packaging plasmids psPAX2 and pMD2.G using Lipofectamine 3000 (Invitrogen, #L3000015) according to manufacturer's instructions. Virus was collected, filtered through 0.22μm filters and used to infect SUM159 cells. Stable cell pools were selected using 1μg/mL puromycin (ACROS Organics).

Re-expression of either codon optimized human Fam3C or CD5-Fam3C sequences into SUM159 shFam3C stable cells was completed using pCDNA3.1 + hygromycin vector versions (Addgene) of the Fam3C inserts listed above, either the native signal peptide (Fam3C amino acids 1-227) or the human CD5 signal peptide (MPMGSLQPLATLYLLGMLVASCLG + Fam3C amino acids 25-227). Primers used for cloning are listed in **Supplementary Table S1.** pCDNA3.1 vectors were originally introduced into cells using Lipofectamine 3000 reagent (Invitrogen, #L3000015) according to manufacturer's instructions. pCDNA3.1 + hygromycin vectors were selected using hygromycin (Invitrogen #10687010); 10ug/ml) to generate stable overexpression cell pools.

Cell count proliferation assay

Cell lines were seeded in a 24 well tissue culture treated plate with three technical replicates at 12,000 cells per well. Cells were trypsinized and counted at 24, 48 and 72 hours after seeding. Three biological replicates were completed, and results were graphed normalized to the mean of the negative control at each time point.

Migration Assay

70uL of a 200,000 cells/mL cell suspension was seeded in each well of an Ibidi Culture-Insert 3 well product (Ibidi, 80369) for 2d migration assays. After 24 hours, the cells were checked to ensure a confluent monolayer, and the insert was removed. Cells were imaged at the 0 hour time point and then allowed to migrate in complete DMEM media described above (DMEM (4.5 g/L glucose, L-glutamine and sodium pyruvate) with 5% FBS, 5% BCS, 1:100 Antibiotic-Antimycotic, 0.05mg Plasmocin prophylactic) for 22 hours, at which point the cells were fixed, stained with crystal violet (Millipore Sigma), and images were acquired at 5x magnification on a Leica DMIL LED microscope with an Amscope MU500 camera and Amscope acquisition software. Cell closure areas were quantified using ImageJ software and calculated as percent wound closed comparing the 0-hour and 22-hour images for each well.

Mammosphere assay

500 cells/ well were seeded for each cell line in a 96 well ultra-low attachment plate (Corning, reference # 3474) in mammosphere media (DME/F12 (Gibco #11330-032); 5ng/mL EGF (Corning #354052), 5ng/mL FGF (Gibco, PMG0035), 1x B27(Gibco, 17504-044). Cells were incubated at 37 degrees Celsius for 10 days. Mammospheres greater than 50μm in diameter were counted. Representative images were acquired at 5x magnification on a Leica DMIL LED microscope with an Amscope MU500 camera and Amscope acquisition software.

Antibodies

The following antibodies were used in this study. Fam3C (Sigma #HPA050548-100ul, Cell Signaling Technology #15171S, Abcam ab72182), PyMT (Abcam ab15085), Ki67 (CST #9129), PERK1/2: P-p44/42 (CST #4370S), IgG antibody (Goat Anti-Human IgG (H+L)-AP Conjugate (BioRad #170-6521), Normal rabbit IgG (Santa Cruz # sc-3888), Normal mouse IgG (Santa Cruz # sc-3878), GM130 (Abcam #169276), CopA (Santa Cruz # sc-398099), CopG (Santa Cruz # sc-393977), GFP (CST #2956P), pStat3 (#9145, Cell Signaling Technologies), Stat3 (#4904, Cell Signaling Technologies), GAPDH (sc-32233; Santa Cruz), HSP90 (sc-13119; Santa Cruz), ATP2A2/SERCA2 (Cell Signaling Technologies; #9580), Vimentin (Cell Signaling Technologies; #5741).

Immunofluorescence

Clean cover slips were coated with poly-L-lysine (Sigma-Aldrich, #P4707-50ml) at a final concentration of 100ug/mL for 15 minutes and washed with Phosphate-Buffered Saline (PBS). Cells were plated onto poly-L-lysine coated coverslips in their normal media. Appropriate samples were treated with 100ng/mL Brefeldin A (Cell Signaling Technology #9972S) for 2 hours before fixation. After 24-48h, cells were washed with PBS and fixed in 1mL of 4% paraformaldehyde (Alfa Aesar) in PBS for 15 minutes at room temperature. Cells were washed 3x in PBS for 5 minutes per wash. Cells were blocked in 1mL of blocking buffer (2% BSA (Fisher, BP9706-100), 0.2% Triton X-100 (Sigma, T-8787), 1mM EDTA in PBS). Primary antibody was added to blocking buffer overnight at 4 degrees (1:200 Fam3C CST cat #15171S, 1:200 GM130 cat# ab169276). Cells were washed in PBS three times for 5 minutes per wash. Secondary antibody (1:1000 Goat anti-Rabbit IgG Secondary Alexa Fluor 488 (Invitrogen A11008) or Goat anti-Mouse IgG Secondary Alexa Fluor 568 (Invitrogen A11031)) was incubated with the cells in blocking buffer for 30 minutes at room temperature. Cells were washed in PBS three times with 5 minutes per wash and dropped onto slides with Dapi containing mounting media (Southern Biotech DAPI Fluoromount-G, cat# 0100-20). Images were taken using an Olympus FV10i LIV laser scanning confocal microscope using a 60x water objective unless otherwise indicated at 37 degrees Celsius. Images were processed, overlayed and areas and intensities were measured using ImageJ software. Fractionation was determined by thresholding GM130 images and particle analysis in ImageJ software.

Immunoprecipitation

Cells were washed in PBS buffer and lysed in 20mM Tris HCl pH 7.5, 150mM NaCl, 1mM EDTA, 1% NP-40 (Anatrace, #NIDP40), 1X Protease Inhibitors (Thermo Scientific, #1861281) for 30 minutes on ice. Samples were spun at 16,000*g for 15 minutes at 4 degrees Celsius to remove nuclei. Supernatant protein concentrations were determined using the Bradford Protein Assay (Bio-Rad cat# 5000006). The required volume of SureBeads ProteinA Magnetic beads (Bio-Rad cat# 161-4013) was thoroughly washed with PBS plus 0.1% Tween 20 (Fisher, #BP337-500). 2mgs of lysate combined with 50uL beads were rotated for one hour at 4 degrees Celsius to pre-clear the lysate. Beads were pre-incubated with antibody as indicated for 30 minutes at room temperature. Pre-cleared lysates were combined with antibody bound resin and incubated end over end overnight at 4 degrees Celsius. Samples were washed 3 times with PBST, resuspended in 30ul of 2x Laemmli reducing denaturing sample buffer, heated to 70 degrees Celsius for 10 minutes, and beads were magnetized to transfer the supernatant to fresh tubes.

Membrane fractionation

Cells were washed in PBS and lysed in 1X Triton X-114 buffer (10mM Tris pH 7.4, 150mM NaCl, 1mM EDTA, 1% Triton X-114 (Sigma-Aldrich #X114-500ml), 1X Halt Protease and Phosphatase Inhibitors (Thermo Scientific, #1861281)) for 30 minutes on ice. Samples were spun at 10,000*g for 5 minutes at 4 degrees Celsius to remove nuclei. Supernatants were transferred to a fresh tube. Lysate was warmed to 32 degrees Celsius for 3 minutes and quickly centrifuged at 10,000 *g for 20 seconds at room temperature. Aqueous

upper phase was transferred to a fresh tube. Aqueous phase was re-extracted by adding 11x Triton X-114 buffer (11% Triton X-114, 10mM Tris pH 7.4, 150mM NaCl, 1mM EDTA, 1X Halt Protease and Phosphatase Inhibitor cocktail (Thermo Scientific, #1861281)). Samples were mixed well and put on ice until solution is clear. Lysate was rewarmed at 32 degrees Celsius for 3 minutes, spun at 10,000 *g for 20 seconds at room temperature and collected upper aqueous phase again. Hydrophobic phase was re-extracted by adding 10-fold volume of 0.06% Triton X-114 buffer, mixed well and put on ice until solution was clear. Solution was rewarmed, spun at 10,000 *g for 20 seconds at room temperature and the aqueous phase was discarded. The volume of membrane extraction solution was increased to 400uL with buffer solution + Halt Protease and Phosphatase inhibitors (Thermo Scientific, #1861281) to volume match with whole cell lysate and soluble fractions. Samples were diluted equal volumes in Laemmli reducing denaturing buffer (60mM Tris-Cl pH 6.8, 1% SDS (VWR, 0227-1KG), 10% glycerol (BDH, BDH1172-1LP), 5% BME (Sigma-Aldrich, M6250-100ml)) and boiled for immunoblotting.

Immunoblotting

As described previously (Woosley et al., 2019), whole cell lysates were prepared by incubating cells in a Tris/Triton lysis buffer (10mM Tris pH 7.4, 150mM NaCl, 1mM EDTA, 1% Triton X-100 (Sigma, #X-100), 1X Halt Protease and Phosphatase Inhibitor cocktail (ThermoFisher; #1861281)) for 30 minutes on ice. Cells were scraped, transferred to an Eppendorf tube and spun at 16,000*g for 15 minutes. Supernatant protein concentrations were determined using the Bradford Protein Assay (BioRad). To isolate protein from conditioned media, cells were serum starved overnight in DMEM (4.5 g/L glucose, L-glutamine and sodium pyruvate) media supplemented 0.1% FBS, 1:100 Antibiotic-Antimycotic (Gibco, #15240-062), 0.05mg Plasmocin prophylactic (InvivoGen, #ant-mpp). Media was collected and spun at 300*g for 5 minutes. Media was transferred to a fresh tube and protein was precipitated using trichloroacetic acid/acetone (Sigma-Aldrich, #T6399-4X100G) for 10 minutes at 4 degrees Celsius. Precipitated protein was isolated by centrifugation at 16,000*g for 5 minutes, washed twice with cold acetone and denatured by incubating at 95 degrees Celsius for 5 minutes with 1× Laemmli reducing denaturing buffer (60mM Tris-Cl pH 6.8, 1% SDS (VWR, 0227-1KG), 10% glycerol (BDH, BDH1172-1LP), 5% BME (Sigma-Aldrich, M6250-100ml)). 1-100μg lysate was resolved on 4-15% gradient gels (BioRad) and transferred to PVDF membrane (BioRad). Membranes were blocked for 1 hour in 5% skim milk in Tris-buffered saline with 0.01% Tween-20 (Fisher, #BP337-500) (TBST) and incubated overnight at 4 degrees Celsius with primary antibody in 5% skim milk/TBST. Membranes were washed in TBST 3 times for 15 minutes each before blocking in secondary antibody diluted in 5% skim milk/TBST for 1 hour at room temperature (Goat anti-Mouse IgG (ThermoFisher; 31430; 1:10,000) and Goat anti-Rabbit IgG (ThermoFisher; 31460; 1:10,000)). Membranes were washed in TBST 3 times for 15 minutes each, incubated briefly in Luminata Forte Western HRP substrate (EMD Millipore), or CDP-Star substrate if AP conjugated (Roche Diagnostics GmbH, #12 041 677 001), and visualized using a CCD camera (BioRad ChemiDoc System; BioRad).

Mass Spectrometry Analysis

Vector control or Fam3C-Fc overexpressing HEK293 whole cell lysates were immunoprecipitated using SureBeads Protein A magnetic beads (Bio-Rad cat# 161-4013) overnight at 4 degrees Celsius. Samples bound beads were thoroughly washed three times in PBS with 0.1% Tween 20 (Fisher, #BP337-500), resuspended in 30ul of 2x Laemmli reducing denaturing sample buffer, heated to 70 degrees Celsius for 10 minutes, and magnetized beads to transfer the supernatants to a fresh tube. Samples were briefly run into a 10% SDS-PAGE gel (BioRad) and a complete sample gel slice was extracted and provided to the Taplin Biological Mass Spectrometry Facility at Harvard Medical School for LC-MS/MS analysis. Proteins with unique and total hits above vector control samples were considered for validation. PantherDB (Thomas et al., 2022) was used to determine Golgi apparatus localized proteins from the list of potential Fam3C interactors.

Cytokine Array

A Proteome Profiler Human Cytokine Array Kit (Bio-Techne, ARY005B) was used according to manufacturer's instructions. For sample preparation, 500,000 human SUM159 cells were seeded on a 6cm tissue culture plate. At 24h post seeding, the plates were washed with PBS and 5ml of low serum media (DMEM with 0.2% FBS, 1:100 Antibiotic-Antimycotic (Gibco, #15240-062), 0.05mg Plasmocin prophylactic (InvivoGen, #ant-mpp)) was added to the cells. 24h later the supernatants were collected and spun down at 500*g for 5 minutes. The supernatants were diluted 1:1 with Array Buffer 6 for a total volume of 1.5mL. The samples were transferred to a pre-blocked membrane and incubated overnight at 4 degrees Celsius on a rocking platform. Membranes were washed three times with 1x wash buffer, probed for one hour on a rocking platform at room temperature with a solution containing 30ul of Detection Antibody Cocktail diluted in a total volume of 1.5ml of 1x Array Buffer 4/6 and washed three times with 1x wash buffer. A 1x Streptavidin-HRP solution was added to the membranes for 30 minutes at room temperature on a rocking platform followed by three washes with 1x wash buffer. Membranes were visualized using a CCD camera (BioRad ChemiDoc System; BioRad).

2D invasion assay

Invasion across a basement membrane was performed using a BioCoat™ Matrigel® Invasion Chamber in Falcon® Companion Plates (Corning; #354480). Matrigel Inserts were rehydrated by adding 500μl of serum free media inside the insert as well as 500μl of serum free media added in the well containing the insert. After 2 hours of rehydration in a humidified tissue culture incubator, 37 degrees Celsius, 5% CO_2 atmosphere, the serum free media was removed and 150,000 cells (in serum free media) per well were seeded in the insert. The insert was then transferred to a new well containing 10% FBS media and placed in a humidified tissue culture incubator. 22 hours later, the media was removed, and the membrane was cleaned with cotton tipped swabs. Invasive cells located on the underside of the chamber were stained with crystal violet (Millipore Sigma; #ECM550) for 10 minutes, followed by two washes in distilled water. Inserts were imaged using a Leica MZ 95 microscope with an Amscope MU130 camera and Amscope acquisition software

Statistical analysis

All statistical analyses were completed using Prism 10 (GraphPad). Results were indicated as mean +/- SEM. Total numbers of biological replicates are indicated in figure legends by n. Total number of technical replicates across all biological replicates are indicated in figure legends by r. Data sets comparing two groups were analyzed using two-tailed unpaired Students *t* test. Data with multiple comparison groups were analyzed using one-way ANOVA with Bonferroni post hoc multiple comparison test. Datasets analyzing multiple groups over time were analyzed using 2-way ANOVA. Simple linear regression and Pearson analysis was used for correlation data. $P<0.05$ was considered statistically significant.

Supplementary Materials and Methods References

Cao, L., C, H., D, C.Z., et al. (2021). Proteogenomic characterization of pancreatic ductal adenocarcinoma. Cell *184*.

Cerami, E., Gao, J., Dogrusoz, U., et al. (2012). The cBio cancer genomics portal: an open platform for exploring multidimensional cancer genomics data. Cancer discovery *2*, 401-404.

Chandrashekar, D.S., Bashel, B., Balasubramanya, S.A.H., et al. (2017). UALCAN: A Portal for Facilitating Tumor Subgroup Gene Expression and Survival Analyses. Neoplasia *19*, 649-658.

Chandrashekar, D.S., Karthikeyan, S.K., Korla, P.K., et al. (2022). UALCAN: An update to the integrated cancer data analysis platform. Neoplasia *25*, 18-27.

Chen, E.Y., Tan, C.M., Kou, Y., et al. (2013). Enrichr: interactive and collaborative HTML5 gene list enrichment analysis tool. BMC bioinformatics *14*, 128-128.

Chung, W., Eum, H.H., Lee, H.-O., et al. (2017). Single-cell RNA-seq enables comprehensive tumour and immune cell profiling in primary breast cancer. Nature communications *8*, 15081-15081.

Consortium, T.G.O., Aleksander, S.A., Balhoff, J., et al. (2023). The Gene Ontology knowledgebase in 2023. Genetics *224*.

de Bruijn, I., Kundra, R., Mastrogiacomo, B., et al. (2023). Analysis and Visualization of Longitudinal Genomic and Clinical Data from the AACR Project GENIE Biopharma Collaborative in cBioPortal. Cancer research *83*, 3861-3867.

Ethier, S.P., Guest, S.T., Garrett-Mayer, E., et al. (2020). Development and implementation of the SUM breast cancer cell line functional genomics knowledge base. NPJ breast cancer *6*, 30-30.

Gao, J., Aksoy, B.A., Dogrusoz, U., et al. (2013). Integrative analysis of complex cancer genomics and clinical profiles using the cBioPortal. Science signaling *6*, pl1.

Gillette, M., S, S., S, C., et al. (2020). Proteogenomic Characterization Reveals Therapeutic Vulnerabilities in Lung Adenocarcinoma. Cell *182*.

Hulsen, T., de Vlieg, J., Alkema, W., et al. (2008). BioVenn – a web application for the comparison and visualization of biological lists using area-proportional Venn diagrams. BMC Genomics 2008 9:1 *9*.

Krug, K., Jaehnig, E.J., Satpathy, S., et al. (2020). Proteogenomic Landscape of Breast Cancer Tumorigenesis and Targeted Therapy. Cell *183*, 1436-1456.e1431.

Lin, E.Y., Jones, J.G., Li, P., et al. (2003). Progression to Malignancy in the Polyoma Middle T Oncoprotein Mouse Breast Cancer Model Provides a Reliable Model for Human Diseases. American Journal of Pathology *163*, 2113-2126.

Minn, A., GP, G., PM, S., et al. (2005). Genes that mediate breast cancer metastasis to lung. Nature *436*.

Ősz, Á., Lánczky, A., and Győrffy, B. (2021). Survival analysis in breast cancer using proteomic data from four independent datasets. Scientific reports *11*, 16787-16787.

Ott, C.M., and Lingappa, V.R. (2002). Integral membrane protein biosynthesis: why topology is hard to predict. Journal of cell science *115*, 2003-2009.

Rao, T., Ranger, J.J., Smith, H.W., et al. (2014). Inducible and coupled expression of the polyomavirus middle T antigen and Cre recombinase in transgenic mice: an in vivo model for synthetic viability in mammary tumour progression. Breast cancer research : BCR *16*, R11-R11.

Streitfeld, W.S., Dalton, A.C., Howley, B.V., et al. (2023). PCBP1 regulates LIFR through FAM3C to maintain breast cancer stem cell self-renewal and invasiveness. Cancer Biology & Therapy *24*.

Tang, W., Zhou, M., Dorsey, T.H., et al. (2018). Integrated proteotranscriptomics of breast cancer reveals globally increased protein-mRNA concordance associated with subtypes and survival. Genome medicine *10*, 94-94.

Thomas, P.D., Ebert, D., Muruganujan, A., et al. (2022). PANTHER: Making genome-scale phylogenetics accessible to all. Protein Science *31*.

Wagner, K.U., Wall, R.J., St-Onge, L., et al. (1997). Cre-mediated gene deletion in the mammary gland. Nucleic Acids Research *25*.

Woosley, A.N., Dalton, A.C., Hussey, G.S., et al. (2019). TGFβ promotes breast cancer stem cell self-renewal through an ILEI/LIFR signaling axis. Oncogene *38*, 3794-3811.
